# Hepialid Moth Diversity in Australia Further Highlighted by Five New Species in the Endemic Genus *Abantiades* Herrich-Schäffer (Lepidoptera: Hepialidae) †

**DOI:** 10.3390/insects17030299

**Published:** 2026-03-09

**Authors:** Michael D. Moore, Mark I. Stevens

**Affiliations:** 1 Earth and Biological Sciences, South Australian Museum, Adelaide, SA 5000, Australia; michael.moore@samuseum.sa.gov.au; 2 School of Biological Sciences, Adelaide University, Adelaide, SA 5005, Australia

**Keywords:** *COI*, barcode, rain moth, taxonomy, unipectinate, rami

## Abstract

We describe five new species of *Abantiades* from Australia, four from Western Australia (WA) and one from south-east Queensland. Of the four WA species, three are from the Geraldton Sandplains biogeographic region (*Abantiades profundus* **sp. nov.**, *A. kolpodes* **sp. nov.**, and *A. patella* **sp. nov.**), and the fourth (*A. lepusaures* **sp. nov.**) was found in the Eastern Goldfields region. The Queensland species (*A. incognito* **sp. nov.**) comes from the south-eastern Queensland biogeographic region. Our five new species demonstrated the importance of wing morphology, genitalia and antennal structure in diagnosing *Abantiades* species. However, antennal structure as a diagnostic character defining these species highlighted historical problems that, in part, were used to erect *Trictena* and *Bordaia*, recently synonymised into *Abantiades*. To explore this further, we used mtDNA *COI* ‘barcode’ sequences and compared our new species with all currently available species for the genus *Abantiades*.

## Simple Summary

Hepialidae is an ancient family with some of the largest and heaviest moths known worldwide. They are an iconic species, often referred to as ‘ghost’, ‘swift’ or ‘rain’ moths, and can be found emerging as adults in their thousands after significant rain. The family is well represented in Australia, and we describe five new species in the endemic genus *Abantiades*. We also further clarify one important taxonomic character for the genus and provide the most up to date DNA ‘barcode’ analysis for all known *Abantiades* species.

## 1. Introduction

Hepialidae Stephens, 1829 (Hepialoidea) is an ancient family with some of the largest and heaviest moths known, with 82 genera and 701 species currently found on every continent except Antarctica [1,2]. The caterpillars and pupae of these moths live hidden during their lifecycle [3], with many being subterranean, and the remainder living within the stems and roots of host shrubs or trees [4,5,6,7]. While South America has 34 genera currently recognised (147 named species), the Hepialidae are currently their most speciose in south-east Asia (9 genera and 193 species) and Australasia (15 genera and 241 species) [2].

The most speciose genus in Australasia is *Oxycanus* Walker, 1856 (78 named species), but in Australia the endemic genus *Abantiades* Herrich-Schäffer, 1855 is currently the largest, comprising 47 named species and including species previously classified in the genera *Trictena* Meyrick, 1890 and *Bordaia* Tindale, 1932 [2,3,8]. *Abantiades* is most diverse across non-tropical Australian regions, where species vary widely in their size, in their antennal form, in the structure of their genitalia, and in their wing pattern, though all have a similar general body habitus. These features are all useful in discriminating species [2,9,10]. In addition to the above morphological features, the mitochondrial *COI* ‘barcode’ gene has also proven extremely useful in distinguishing between *Abantiades* species (e.g., [8,11,12,13,14]).

Species in the genus *Abantiades* are notable for mass emergences of adults triggered by rain events. There is evidence to suggest that the caterpillars in this genus feed on fungi and lichen in leaf litter after hatching but then dig into the ground, where some re-surface regularly to feed on herbs or grasses and others remain buried, feeding on or in the roots of their host plants [3,7,15]. Adult hepialids emerge for mating and egg laying, and these emergences can occur in the thousands, coinciding with rain fronts that sweep across Australia [5,6,16]. Usually, these fronts occur in Autumn, but for the last few years (longer in Western Australia) it has been much drier with more substantial rainfall coming later in Autumn or early Winter when the nights are significantly colder; how this will affect the life cycles and survival of these moths is unclear. In situations where rainfall is particularly scarce or infrequent, such as in central Australia, emergence can be limited to a single night [5,17].

This reliance on rain for adult emergence, however, has created uncertainty on accurate species ranges, and much of what is known is based on historical, and often sporadic, records over time. For example, such distribution data has been important for exploring pre- and post-fire species ranges on Kangaroo Island for all hepialid species, comparing new records to historic, but most species distributions were based on rare occurrence records (see [13]). Furthermore, this rareness of specimens has also influenced biological and taxonomic information, for example *A. paradoxa* (Tindale, 1932) males were collected prior to 1932, but the first female specimen was only collected in 2014 [8]. For these reasons, particularly in locations with poor records, targeted collections are crucial. On 4 June 2023, a rain event in the Geraldton Sandplains biogeographic region near Kalbarri in Western Australia, a region with few observations, resulted in thousands of hepialid adults emerging. Targeted collections at the time identified three known *Abantiades* species, *A. kayi* Moore and Beaver, 2020, *A. neglecta* Simonsen, 2018, and *A. argentata* (Tindale, 1932). A second collection (5 June 2023) identified seven hepialid species that included three in the genus *Fraus* Walker, 1856, *A. kayi*, and three undescribed *Abantiades* species.

Here, we describe five new species from the genus *Abantiades*. Three new species are those mentioned above from the Geraldton Sandplains biogeographic region, and a fourth is from specimens collected in 2007 in the Goldfields region of Western Australia. Our fifth new species was originally identified as *A. atripalpis* (Walker, 1856) collected from south-eastern Queensland, but was identified by us as a new species. These five new species will raise the number of described *Abantiades* in Australia from 47 to 52 species. For the new species here, one has tri-forked rami, two have un-forked rami and two fit into the bi-forked category (see [8]). This structure of the rami on the antenna was one of the characters originally used to erect the two genera ‘*Trictena*’ and ‘*Bordaia*’ that were synonymised with *Abantiades* [1], recently supported by a limited mtDNA *COI* study of 34 *Abantiades* species [8]. To further explore our new species, here we expand on the mtDNA ‘barcoding’ to include all currently available species within the genus *Abantiades*.

## 2. Materials and Methods

### 2.1. Specimen Sampling

**Figure 1:**
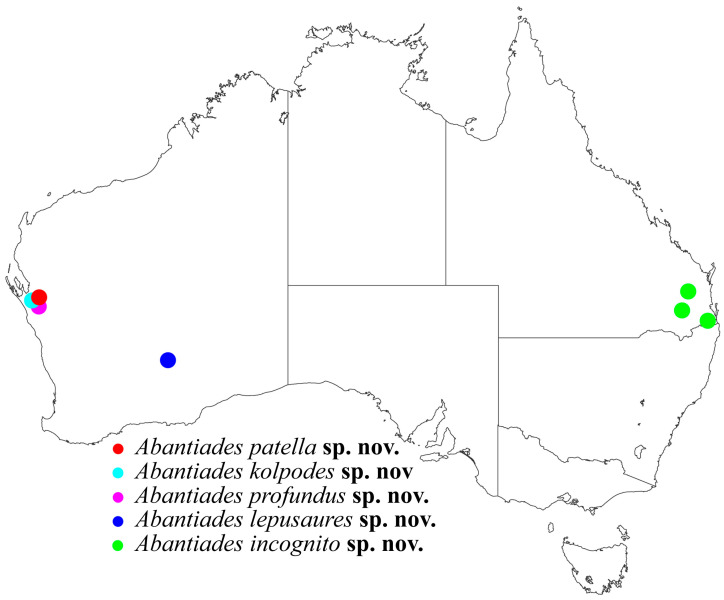
Map of Australia showing the known distribution of the five new species of *Abantiades*.

All specimens collected from the Geraldton Sandplains biogeographic region in Western Australia on two evenings (4 and 5 June 2023) were included. Specimens collected in 2007 in the Goldfields region of Western Australia were also included. In addition to these Western Australian collections, we had the opportunity to examine three specimens originally identified as *A. atripalpis* from the Queensland Museum that were collected in Queensland between 1943 and 1972 (Figure 1). Table S1 details all specimens used, in particular Museum registration numbers, GenBank accession numbers, and details of locality information. We did not have access to specimens or sequence data for *A. mysteriella* (Simonsen, 2018), and we were not able to generate useful sequence data for *A. moesta* (Tindale, 1932) or *A. incognito* **sp. nov.**

### 2.2. Molecular Analyses

DNA extraction and PCR amplification were completed at the South Australian Regional Facility for Molecular Ecology and Evolution (SARFMEE) from a single leg from each specimen. DNA was extracted using a Gentra Puregene^®^ DNA Purification kit (Gentra Systems Inc., Minneapolis, MN, USA) according to the manufacturer’s protocol, and amplified gene fragments were sent to Macrogen Inc. (Seoul, Republic of Korea) for purification and sequencing. All other sequences were amplified using the universal mitochondrial DNA (mtDNA) cytochrome *c* oxidase subunit I (*COI*) gene ‘barcoding’ primers (LepF1/LepR2, LCO1490/HCO2198) [18,19] or using single-molecule real-time sequencing (SMRT) [20] in the PacBio Sequel platform (Pacific Biosciences, Menlo Park, CA, USA) at the Canadian Centre for DNA Barcoding (CCDB), University of Guelph, Ontario, Canada.

All new *COI* sequences generated by us were inspected to resolve ambiguous base calls and checked for potential contamination using BlastN (NCBI; https://blast.ncbi.nlm.nih.gov/Blast.cgi, accessed on 20 February 2025). The resulting sequences were aligned with previously generated (and publicly available) *COI* sequences in Sequencher version 5.1 (Gene Codes, Ann Arbor, MI, USA) to produce a 658 bp (*COI*) alignment of 58 sequences that included an ingroup of 49 *Abantiades* species and five outgroup species (Table S1; and see Data Availability statement). PAUP* ver. 4.0a169 [21] was used to obtain sequence divergence values among species using uncorrected patristic distances with all three codon positions (Supplementary Materials Table S2). Maximum likelihood (ML) trees were generated using the substitution models (1st—TIM + F + I + Γ, 2nd—HKY + F + I, 3rd codon—TVM + F + Γ) calculated from the IQ-TREE web server [22], with 10,000 ML (ultrafast) bootstrap replicates [23]. The ML tree was visualised using FigTree ver. 1.4.4 (https://beast.community/figtree, accessed on 20 February 2025) and modified using Adobe Illustrator version 30.2 (Adobe Systems, Inc., San Jose, CA, USA).

### 2.3. Morphological Analyses

**Figure A1:**
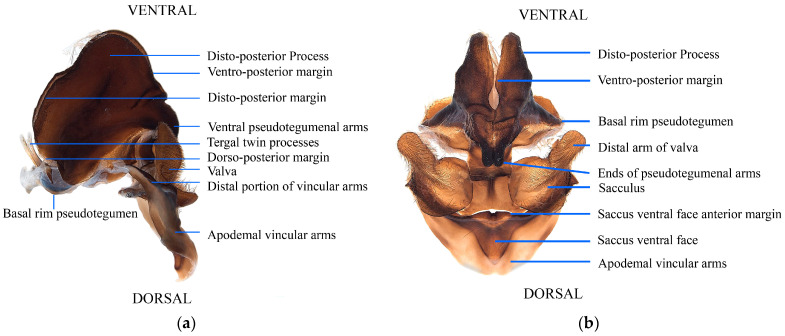
(**a**) Lateral and (**b**) anterior views of the male genitalia of *Abantiades atripalpis* with labelled diagnostic terminology used in the descriptions of the five new species of *Abantiades*. Terms follow that of Dugdale [25] and Simonsen [1].

**Figure A2:**
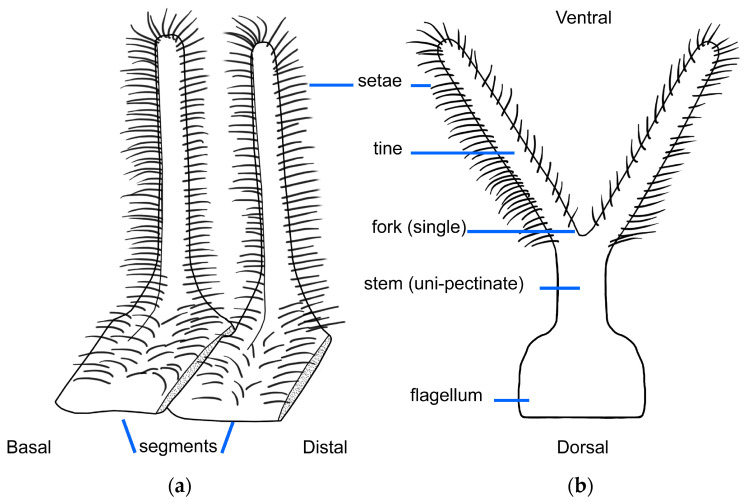
(**a**) Lateral view of rami, and (**b**) looking distally, rami in basal half of flagellum. The antennal illustration with labelled diagnostic terminology used in the descriptions of the five new species of *Abantiades*.

**Figure A3:**
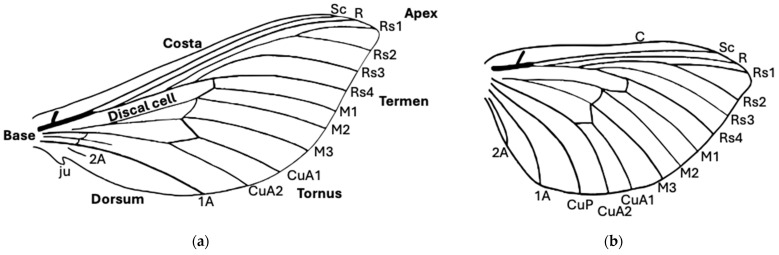
(**a**) Forewing and (**b**) hindwing with labelled diagnostic terminology used in the descriptions of the five new species of *Abantiades*. Figure adapted from Simonsen [1].

Three primary diagnostic characters are used to identify *Abantiades* species, genitalia structure (see Appendix A—Figure A1), antennal structure (Figure A2), and wing morphology (Figure A3), with the latter being problematic for a number of species (e.g., *A. labyrinthicus* (Donovan, 1805), *A. mcquillani* Simonsen, 2018, and *A. rubrus* Moore and Beaver, 2019). For the genitalia, the pseudotegumen is a significant structure and is the most important character separating *Abantiades* species (see Figure A1 for terms). All *Abantiades* have long antennae with between 53 and 74 segments. Each antennal segment possesses a single ventrally projected stem arising from each flagellum (or filament) [1]. The arrangement of stems, in a comb like projection, from the flagellum is termed pectination, and each projection is termed a ramus (or rami). Because only a single stem erupts from each segment in *Abantiades*, they are termed unipectinate; in some species, the stem forks once or twice to produce bi-forked and tri-forked species; others do not fork and are termed un-forked, though the ramus might distally be intricately shaped (see Figure A2 for terms). Initially, the amount of forking (wrongly diagnosed as ‘pectination’) was thought to be a taxonomic character defining genera by Meyrick [24] and later by Tindale [5], and Simonsen [1] questioned this interpretation (but retained the terminology) and synonymised the two genera ‘*Trictena*’ and ‘*Bordaia*’ with *Abantiades*.

All dissections involved removing the abdomen or posterior of the relevant specimen, placing the sectioned material in 10% KOH solution and heating in a boiling water bath for between 5 and 15 min depending on the condition of the specimen. All soft organs and tissues were removed, leaving the abdominal tube and the sclerotized parts of the reproductive organs. These were imaged in an alcohol bath using a Leica imaging suite comprising a Leica binocular microscope, Leica DFC 500 camera (Leica Microsystems, Wetzlar, Germany) with the LAS X Core software version 3.09. Whole specimen images were taken using a Canon EOS camera (Canon Inc., Tokyo, Japan), with a Canon EF 100 mm 1:2.8 Macro lens and Canon Speedlite Transmitter and Speedlite 430 EX11 flashes. The images were then stacked using the Zerene stacker version 1.04 (Zerene Systems LLC, Richland, WA, USA). All data label references are verbatim, and morphological terminology follows that of Dugdale [25] and Simonsen [1]; for comparisons with previous hepialid descriptions, the genitalia have been displayed in figures (and labelled) with ventral structure upwards in the image and dorsal structure downwards (see Figure A1).

## 3. Results

### 3.1. Molecular Analyses

**Figure 2:**
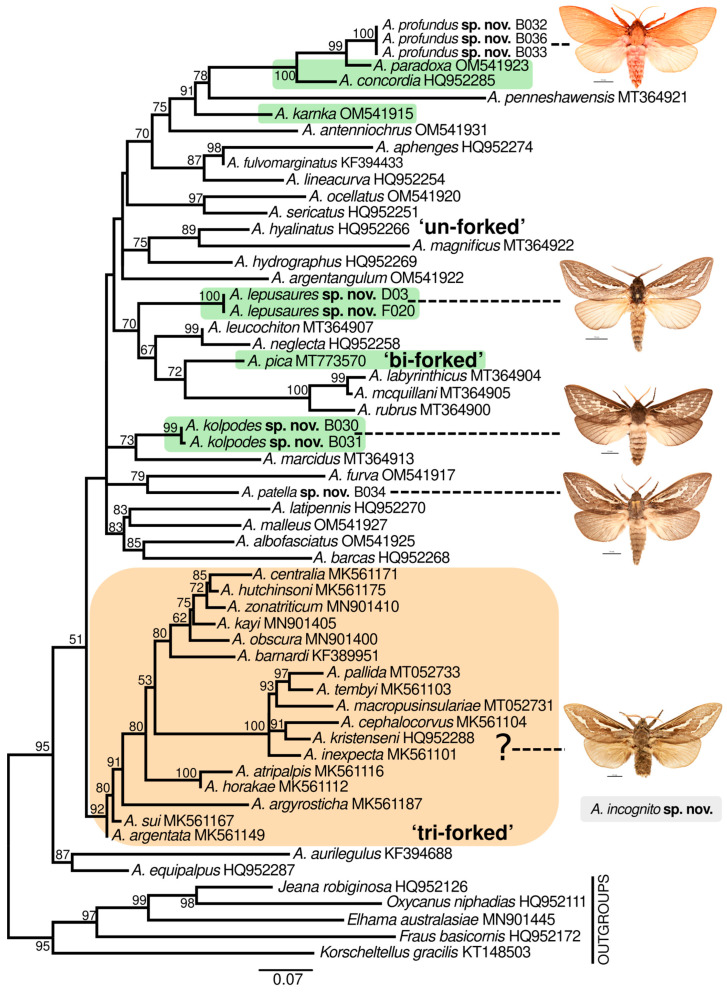
Maximum likelihood tree (midpoint-rooted) using the *COI* gene for 53 *Abantiades* sequences from 49 species and five outgroup taxa. Bootstrap support values above 50% are shown. Brown box indicates all ‘tri-forked’ species and green boxes all ‘bi-forked’ species; all other species are ‘un-forked’ (see also Table 1). We did not have sequence data for three species—*A. moesta*, *A. mysteriella*, and *A. incognito* **sp. nov.** A close (morphological) affiliation of *A. incognito* **sp. nov.** with *A. inexpecta* in the ‘tri-forked’ clade is proposed. Details for all new sequences and those obtained from GenBank are detailed in Table S1, and a checklist of all *Abantiades* species is shown in Table 1.

The ML analysis for the 49 *Abantiades* species returned a well-supported monophyletic clade (midpoint rooted) (Figure 2) with sequence divergences of *Abantiades* species from the five outgroup taxa ranging from 5.9% and 14.5% (Table S2). This comparison with the 49 defined *Abantiades* species supports the separation of our four new species (see Section 3.2 below for comparison to the full 52 species, and Table 1). Each ingroup *Abantiades* species is well defined, with sequence divergence values generally ranging from 2.2% to 13.4% (Table S2). These distances are similar to those reported elsewhere using the same *COI* ‘barcode’ gene region for moths [26,27,28,29] and for hepialids, and have been shown for some species comparisons to be less than 2% (e.g., [8,12,30,31]). For example, pairwise comparisons between *A. horakae* Simonsen, 2018 and *A. atripalpis* is 1.6%, and between *A. zonanatriticum* Moore & Beaver, 2020 and *A. hutchinsoni* Moore & Beaver, 2020 are 1.8%, but can be as low as 0.9% between *A. sui* Simonsen, 2018 and *A. argentata* (Figure 2; Table S2).

Sequence divergences among the new *Abantiades* species and their nearest congeneric neighbours ranged from 2.9% to 11.8% (Table S2). Comparisons of sequence divergences between the ‘un-forked’ species *A. profundus* **sp. nov.** ranged up to 13.4% from all *Abantiades* species included here, with a 2.9% divergence from its closest neighbour *A. paradoxa* (a ‘bi-forked’ species). Similarly, sequence divergences of our second ‘un-forked’ species *A. patella* **sp. nov.** ranged up to 11.1% among species, but 6.4% from its closest neighbour, also an ‘un-forked’ species, *A. furva* (Tindale, 1932). The sequence divergences of *A. kolpodes* **sp. nov.** (‘bi-forked’ species) varied up to 11.6% and by 4.6–5.0% for its closest neighbour, an ‘un-forked’ species, *A. marcidus* (Tindale, 1932). The sequence divergences of the fourth new WA species from further south in Coolgardie, a ‘bi-forked’ species, *A. lepusaures* **sp. nov.**, range up to 11.9% and 4.7–4.8% for its closest neighbour, an ‘un-forked’ species, *A. leucochiton* (Pfitzner, 1914).

We were not able to retrieve useful *COI* sequences from the Queensland new species *A. incognito* **sp. nov.**, but based on morphology it has a close allegiance to *A. inexpecta* Simonsen, 2018 (Figure 2) (see Section 3.2 below). All these species are united morphologically (structure of the rami on the antenna) and by *COI* sequences (where available) in a well-supported monophyletic ‘tri-forked’ clade (Figure 2). By comparison, *A. paradoxa* and *A. karnka* (Tindale, 1941), both ‘bi-forked’ species, are united in a well-supported clade with the ‘un-forked’ *A. penneshawensis* (Moore & Beaver, 2021) and now joined by *A. profundus* **sp. nov.**, which is also ‘un-forked’. Other ‘bi-forked’ species in our analysis also do not align with other ‘bi-forked’ species, such as *A. pica* (Tindale, 1932) and the two new species *A. lepusaures* **sp. nov.** and *A. kolpodes* **sp. nov.** (Figure 2). We note some caution with low-support nodes that will require further study with additional genomic markers, but more support is present for these internal nodes.

### 3.2. Systematics

**Family Hepialidae Stephens, 1828**

Type species: *Phalaena humali* Linnaeus, 1758 (by original designation)

**Genus *Abantiades* Herrich-Schäffer, [1855]**

Type species: *Epiolus hyalinatus* Herrich-Schäffer, 1853 (by original designation)

Included species: Grehan et al. [2] provides a detailed catalogue and checklist for the family Hepialidae, that includes all 47 *Abantiades* species, and Table 1 provides a checklist for all 52 *Abantiades* species.

**Diagnosis**

We follow the diagnosis for the genus *Abantiades* by Simonsen [1] (p. 54). In particular, “The patch of elongate scales at the forewing base and the ‘sensory tubercles’ on S2 are both unique and diagnostic for *Abantiades*”. We also note that they have hepialine wing venation [25,32], and unipectinate antennae (with either un-forked, bi-forked or tri-forked rami) that most usually lack scales on the flagellum (except for some basal segments). An exception to this is *A. mysteriella* with bipectination and scales [1].

#### 3.2.1. ***Abantiades patella* sp. nov.** (Figure 3, Figure 4 and Figure 5)

urn:lsid:zoobank.org:act:45FF56C9-1405-4716-9958-BB0BF8D9BBB7

***Material Examined***
***Holotype***
  In WAM. ♂. **West. Aust.**, Geraldton Sandplains | Kalbarri National Park | Kalbarri. 27.7164° S, 114.3244° E | 5 June 2023; M&M Moore. || Spec. No | 23181 | Leg removed | for tissue | storage | MD Moore.

***Diagnosis***

The male appears grey-brown in colour with two large linear white marks on forewing. Hindwing brown-grey, large buff-white patch covering posterior wing axil (where dorsum meets thorax), abdomen, and part dorsum.

The unique shape of antennal pectinations identifies the male of this species. On basal half of antennae, rami shaped like oblate ellipses, orientated upright along flagellum. *Abantiades leucochiton* and *A. neglecta* have a similar ramal arrangement (un-forked) but in these species the rami are taller and more circular, and they have white hindwings, opposed to brown in this species. The overall shape of the male genitalia is most similar to *A. aurilegulus* Tindale, 1932 and *A. equipalpus* Moore, 2014, but in those species the dorsally directed pseudotegumenal arms have very narrow, pointed ends, whereas in *A. patella* **sp. nov.** they are large, thick and bulbous. The brown colour of the fore- and hindwings are most similar to *A. equipalpus*, but in that species there is only a single, long, white line on the forewing, instead of the two distinctly separated white marks on *A. patella* **sp. nov.**

The mtDNA COI ‘barcode’ sequences are available from GenBank (https://www.ncbi.nlm.nih.gov/genbank/; lodged 5 March 2026) for the holotype B034 (23181; accession number PZ098962).

***Description***

**Figure 3:**
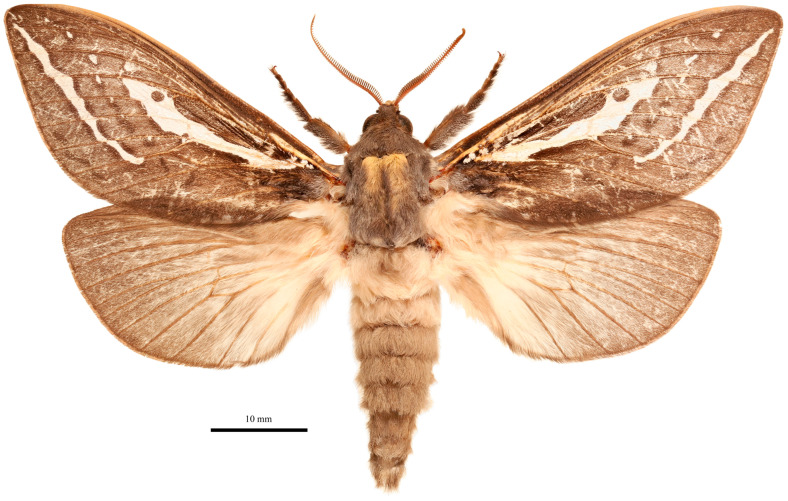
*Abantiades patella* **sp. nov.**, habitus of male holotype, dorsal view. ‘Geraldton Sandplains | Kalbarri National Park | Kalbarri. 27.7164° S, 114.3244° E | 5 June 2023; M&M Moore’.

***Male*** (Figure 3)

*Head*

Two large compound eyes dominate head capsule. Crown densely covered in upright, long, blunt ended, linear, dark grey, scales, except around antennal sclerite where, ring of yellow scales. Fronto-clypeal area covered as per crown.

**Figure 4:**
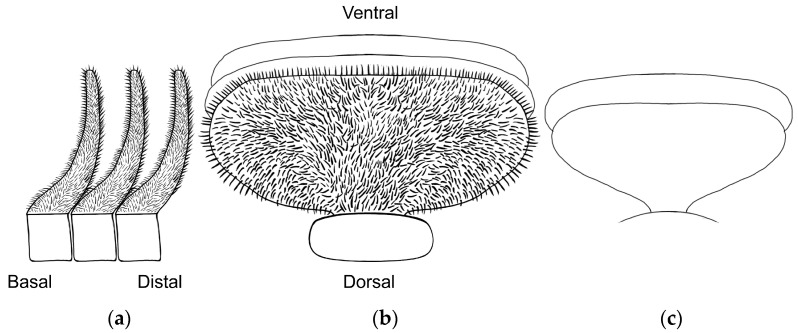
Antennal rami of male *Abantiades patella* **sp. nov.** (**a**) Lateral view of rami, (**b**) looking distally, rami in basal half of flagellum, and (**c**) looking distally, rami in distal half of flagellum.

Antenna (Figure 4): 57 segments between one-third and one quarter length costa. Upper side flagellum pale, underside and rami dark brown. Each segment is unipectinate and un-forked. Rami rapidly expand laterally after erupting from stem. Shape of rami vary along flagellum length; basal, two-thirds of the length oblate ellipsoids, then become fan shape, distally thick linear. Largest rami found one-third along flagellum length. Ramal plates closely packed, along flagellum; covered in small, colourless, setae particularly around edges (Figure 4). Very sparse, yellowish scales adhere to first three segments of antenna.

Labial palps: Three segmented, middle slightly longer than basal, both cylindrical with widened distal ends. Distal segment smallest, spherical. All densely clothed in mixture of dark yellow straw and light grey linear scales tightly appressed to palps.

*Thorax*

All densely covered in long piliform scales. Dorsal: pro-thorax dark grey, meso yellow straw, meta dark grey, laterally all segments grey. Ventral: anterior half grey, posterior light grey. Laterally, white.

Forewing, 35 mm. Veins straw coloured, emphasised by accumulated scales. Membrane whitish, transparent. Dorsal: Costa densely covered in small lanceolate scales of straw or grey colour. C to Sc densely covered, basally dark grey spathulate that become much smaller, lanceolate and yellow grey as move towards apex. Most of the rest of wing covered in obtuse round ended leaf shaped scales in shades of grey to brown/cinnamon colouring. Posteriorly the scales become more linear lanceolate, towards wing base more piliform. Two large and distinct white marks: first, subterminal, from just short of apex to CuA1; irregular, linear, filled with shining white, lanceolate, scales, strongly bordered in dark cinnamon grey; second, discoidal, more substantial, slightly crescentic, irregular, filled lanceolate, shining, white scales. Posterior to discoidal line, patches of dark, cinnamon grey scales making disjointed border. Anterior margin not bordered until in basal region where, dark brown scales border white mark anteriorly and posteriorly. A post medial line of white, dark cinnamon grey edged spots, and dark cinnamon grey patches stretching from apex to CuA2. Terminal line of dark cinnamon grey patches in region of tornus. Patch of long, linear grey scales at wing base and patch white piliform scales in posterior wing axil. Wing fringe of golden spathulate shaped scales runs from apex to CuA1 then grey, linear, then whitish, piliform to wing axil. Ventral: Costa, densely covered in small, lanceolate, yellow-straw coloured, scales. C to Sc, basal area mixture of light grey lanceolate and piliform scales which moving towards apex become lanceolate, straw yellow. Sc to R, basally mid to dark grey piliform, moving towards apex become shorter, sparser and lighter in colour; at apex, down termen edge to M3, longish lanceolate cinnamon coloured. Inside this area, scales replaced by sparse covering of straw coloured piliform scales except in discoidal area and wing axil where scales distinctly denser and whiter.

Hindwing, 27 mm. Dorsal: Costa, densely packed light grey linear scales. Region C to R light grey-straw. Outer third wing sparsely covered in long linear lanceolate dark cinnamon grey scales, which towards wing base become linear then piliform. Basal one fifth of wing densely covered in whitish buff piliform scales. Ventral: Costa as above, C to R densely packed the small light grey lanceolate scales, some at base piliform. Outer one third wing plus apex moderately well covered in long lanceolate scales. Inner two thirds become piliform, anterior of M2 straw coloured; posterior all white.

Legs. Foreleg/midleg: Dorsally densely covered in mixture of linear, mid grey and straw coloured scales. Laterally long linear, white tipped, grey scales. Ventrally grey straw except for tarsi, densely covered in short, linear, yellow straw scales. Hindleg: All yellow straw, light grey laterally. Epiphysis, vase shaped with strong point on outer margin. Arolium narrow sub elliptical, wider end basal.

*Abdomen*

All densely covered in long piliform scales. Dorsal: T1 and T2 white, T3 slightly greyer, T4 to T9 light grey. Ventral: light grey.

**Figure 5:**
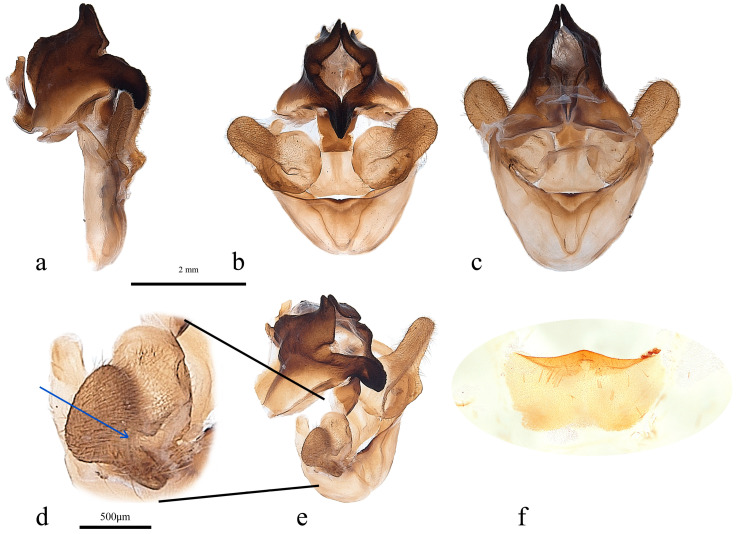
*Abantiades patella* **sp. nov.**, male genitalia ((**a**–**e**): ventral top; dorsal bottom): (**a**) lateral, (**b**) anterior, (**c**) posterior, (**d**) enlarged image of valva showing cleft, (**e**) anterio-lateral, and (**f**) sternite 8 (posterior top; anterior bottom).

Sternite 8 (Figure 5f). Posterior margin concave but with well-defined raised central point. Margin seems folded with extra sclerotising. Lateral margins concave, narrowing anteriorly. Anterior margin sinuous.

Genitalia (Figure 5a–e). Vincular arms join broadly at apices whence widen rapidly reaching maximum at one third of length; then reduce to produce two short distal arms that curve towards midline. The arms produce a ‘V’ shaped saccus with obtuse rounded dorsal apex. Ventral margin on anterior face sinuous, with shallow, more strongly sclerotised central notch. Posterior face more membranous with large central ‘V’ shaped invagination (Figure 5c).

Pseudotegumen angular, deep as long. Dorso-posterior margin long, gently concave (Figure 5a). Posterior corner broad based, convex meeting essentially straight disto-posterior margin in slight convexity. The disto-posterior projection long, narrow, and pointed, with an obtuse rounded apex (Figure 5a). Disto-ventral margin rises steeply, making forward-facing angled point before dipping into deep rounded concavity where it meets pseudotegumenal arms. Ventral margin arms somewhat irregular, rising out of concavity, bends and runs straight producing broad rounded convexity as it meets dorsal margin with wide but shallow point (Figure 5a). Anterior portion of pseudotegumenal rim long, slightly concave adhering closely to blade shaped intermediate plate (Figure 5a). Rear of pseudotegumenal rim widish and angles downwards producing wide base for substantial chitinous and sclerotised strap like twin processes. Ventral portion of pseudotegumen and its arms more heavily sclerotised.

Valva (Figure 5d,e) large, having rounded sacculus; distal arms broadly based with broad apices. Ridge runs down face of distal arms onto upper part sacculus producing deep cone shaped depression in faces of sacculus and distal arm; accentuated by distinct forward-facing lip in dorso-lateral corner. Distal arms well covered in lengthy setae.

Trulleum somewhat tall, trapezoid. Juxta, trapezoid though much squarer (Figure 5b).

***Remarks***

Only one male specimen was collected. No female specimen is available at this time. MtDNA *COI* ‘barcode’ sequence from holotype unique, and places *A. patella* **sp. nov.** in a clade with *A. furva* (Figure 2). Figure 302 in Simonsen [1], the genitalia of *A. furva* is illustrated showing the valva with a depressed area in the upper part of the sacculus much like the valva of *A. patella* **sp. nov.** (Figure 5b,d,e).

***Distribution***

Collected from the Geraldton Sandplains biogeographic region in Kalbarri National Park on 5 June 2023, in semi-arid, low density, low height woodland (see Figure 1).

***Etymology***

The Latin for platter (ellipsoid plates used for serving food) is patella. The antennal rami of this species remind us somewhat of those objects.

#### 3.2.2. ***Abantiades kolpodes*** **sp. nov.** (Figure 6, Figure 7 and Figure 8)

urn:lsid:zoobank.org:act:BD99B1A2-EBB6-4DE3-8F79-0386E4792925

***Material Examined***
***Holotype***
  In WAM. ♂. West. Aust., Geraldton Sandplains | Kalbarri National Park | Kalbarri. 27.7164° S, 114.3244° E | 5 June 2023; M&M Moore. || Spec. No | 23177 | Leg removed | for tissue | storage | MD Moore.
***Paratype***
  In SAMA. ♂. West. Aust., Geraldton Sandplains | Kalbarri National Park | Kalbarri. 27.7164° S, 114.3244° E | 5 June 2023; M&M Moore. || Spec. No | 23178 | Leg removed | for tissue | storage | MD Moore ||| SAMA No.31-21862.

***Diagnosis***

Smaller species possessing dark grey forewing with three well marked silvery white lines, white jugal areas and no scrolling. Hindwing unpatterned, greyish-white with veins defined. Head, anterior part thorax dark grey, white posterior, abdomen anterior white, posterior grey. Antennae bi-forked and golden yellow.

The above features are enough to distinguish this species from all other *Abantiades* species however the intricate and highly folded pseudotegumenal margin in genitalia is also unlike any other *Abantiades* species. In appearance *A. kolpodes* **sp. nov.** is most similar to *A. pica*; both species being smallish and bi-forked but *A. pica* has grey antennae with filamentous tines whereas *A. kolpodes* **sp. nov.** has golden yellow, non-filamentous antenna. *Abantiades antenniochrus* Moore, 2014 also has yellow antennae but with un-forked rami. *Abantiades kolpodes* **sp. nov.** with grey forewing and white hindwing also differs from *A. antenniochrus* with brown fore- and hindwings. *Abantiades albofasciatus* (Swinhoe, 1892) and A. neglecta are also found in this location but they have brown, un-forked antennae.

The mtDNA *COI* ‘barcode’ sequences are available from GenBank (https://www.ncbi.nlm.nih.gov/genbank/; lodged 5 March 2026) for the holotype B030 (23177; accession number PZ098960) and paratype B031 (23178; PZ098961).

**Figure 6:**
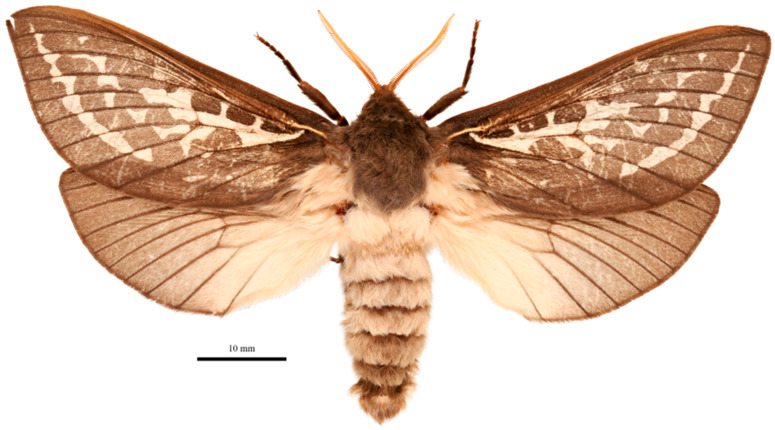
*Abantiades kolpodes* **sp. nov.**, habitus of male holotype, dorsal view. Geraldton Sandplains | Kalbarri National Park | Kalbarri. 27.7164° S, 114.3244° E | 5 June 2023; M&M Moore.

***Description***

***Male*** (Figure 6)

*Head*

Two large compound eyes dominate the head capsule. Fronto-clypeus densely covered in long, linear, dark cinerous brown scales except for a very narrow ring of yellow-straw scales around antennal sclerite. Crown densely covered in long upright linear dark grey scales.

**Figure 7:**
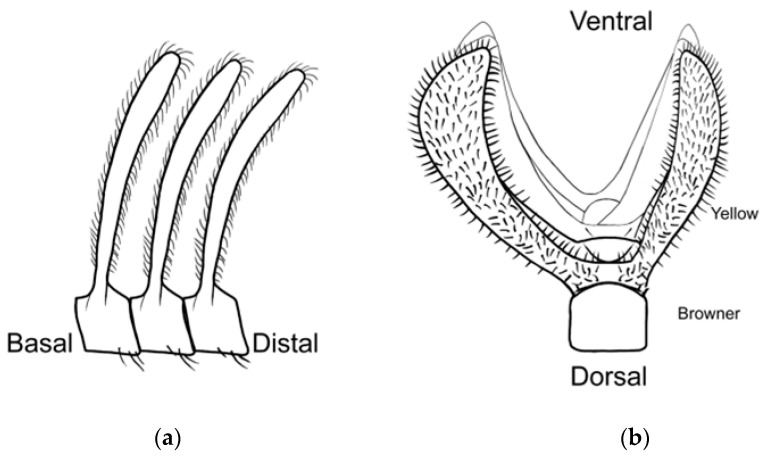
Antennal rami of male *Abantiades kolpodes* **sp. nov.** (**a**) Lateral view of rami, and (**b**) looking distally, rami in basal half of flagellum.

Antennae: Bi-forked rami, 53 segments, one quarter length of costa. Segments barely unipectinate, with fork’s tines emanating from the stem corners (Figure 7). All coloured golden yellow. Rami change shape along length, maximum length reached segment 12. Slight ramal lean to distal end, proximal and distal faces flat. Tines different shapes. Anterior wider, more shapely, posterior almost parallel sided, constant width. Along basal half of antenna inner margins both slightly concave. Anterior tines’ outer margins varying convexity, creating tines of constantly changing width, widest near the tine’s apex, ending in blunt, rounded point. Posterior tine curve, more constant and regular in shape. In distal half antenna, tines more linear, similar in form. Flagellum segments circular basally but become dorso-ventrally flattened distally becoming more plate like. Rami all covered, in short fine colourless setae, with a very few longer stiffer setae on flagellum segments. A few short blunt ended yellow-straw scales present on antennae up to segment 4.

Labial palps: Three segmented, porrect, distal segment smallest, orbicular, middle, longest, cylindrical 1.5× to 2× longer than cylindrical basal segment, all densely covered in fine linear scales tightly appressed to palps, brown with golden sheen, particularly distal segment where circular scale arrangement enhances effect.

*Thorax*

All densely covered in longish piliform scales, laterally slightly longer. Dorsal: Pro-thorax dark grey, meso-thorax, light grey, meta-thorax, white, lateral scales white. Ventral: Anterior grey, posterior white, laterals white.

Forewing, 38 mm. Dorsal: Veins white, membrane quite transparent. Costa and Sc both densely covered in lanceolate scales, buff coloured apically, grey-brown basally. C to Sc space densely filled with brown-dark grey spathulate scales. Most of wing sparsely covered, in brown-grey or shing white, blunt ended, leaf shaped, scales which lengthen and become piliform near base. Three white lines present; subterminal band from forward Rs2 to past CuA1, regular, lens shaped; discoidal band starts M vein origin, along vein to 60% distance to termen, less regular shape, some dark grey highlighting anterior to mark blending in colour with the C to Sc space; post discal line irregular, more series irregular white spots. Some white piliform scales near rear wing axil with body. No scrolling. Ventral: Markings and scales visible through membrane. Costa densely covered in linear lanceolate scales, grey basally, buff distally. C to Sc space similar colour, scales more piliform basally. Termen and apical area sparsely covered in longer lanceolate grey scales. Rest, very sparsely covered in piliform scales, longer basally, grey and grey-white forward of 1A, white posteriorly.

Hindwing, 30 mm. Dorsal: Unpatterned, only marginally lighter than forewing. Costa and other veins covered in linear lanceolate, dark grey-brown scales, highlighting them. Area anterior CuA1 sparsely covered in grey scales, shorter, more linear near termen, lengthen to piliform near base. Posterior CuA1 scales linear whitish buff, piliform basally. Ventral: As for forewing but more white area basally.

Legs. Foreleg/midleg: Dorsal coxa densely covered in dark grey, longish, hairlike, scales: Ventral grey. Femur dorsal grey, ventral light grey. Tibia dorsal grey ventral mixture straw and grey, laterals dark grey. Epiphysis, relatively smaller than usual, more slit like, with thick covering of grey scales. Tarsus dorsally longish grey scales, ventrally densely covered in short, fine, linear, golden-straw, scales. Hindleg: Femur and tibia dorsally covered in longish grey-brown scales ventrally mixture grey and straw. Tarsus mixture grey and straw.

Arolium colourless, ellipsoid, broader basally.

*Abdomen*

Dorsal: Covered in long piliform scales. Tergite 1 white, tergites 2-6 white becoming greyer, tergites 7-8 brown-grey. Ventral: Sternite 1 white-grey, 2-7 grey to dark grey, 8 light grey, anterior white fringe to genital opening.

**Figure 8:**
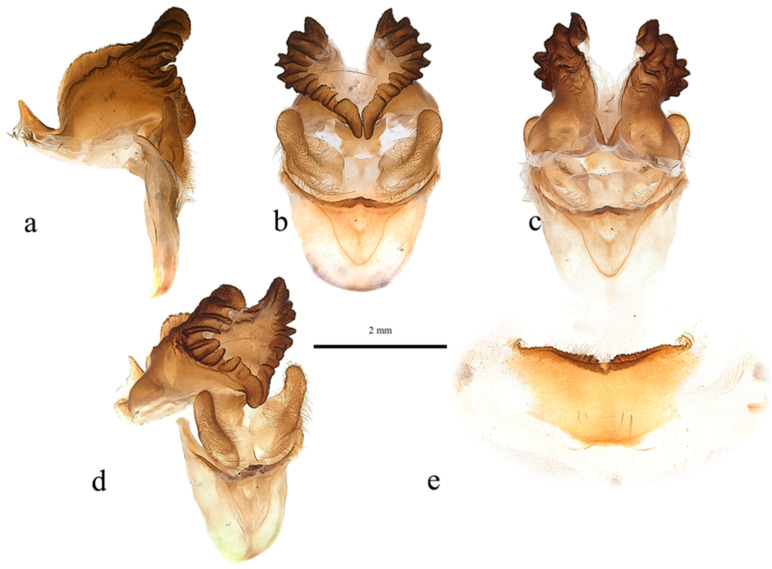
*Abantiades kolpodes* **sp. nov.**, male genitalia ((**a**–**d**): ventral top; dorsal bottom): (**a**) lateral, (**b**) anterior, (**c**) posterior, (**d**) antero-lateral, and (**e**) sternite 8 (posterior top; anterior bottom).

Sternite 8 (Figure 8e). Complex, Posterior margin 3× wider than anterior. Sides, moving anterior, angled towards midline. Posterior margin concave, sinuous with a deeper central concavity, sclerotised reinforcement along entire width, minutely toothed with defined central invagination; post marginal fold present, strongly sclerotised. Anterior margin straight.

Genitalia (Figure 8a–d). Vincular arms meet broadly producing rounded prominence. Outer margin, convex proximally, straight distally. Inner margin runs parallel basally laterally producing a convexity and narrows width. Laterally, apodemal vincular arms thin and flat. Sacculus internally ‘V’ shaped with rounded obtuse apex (Figure 8b,c). Ventrally widens to double width. Ventral margin of anterior face of saccus sinuous with central point. Entire margin more heavily sclerotised (Figure 8b,d). Ventro-lateral arms of saccus extend extensively laterally and ventrally adhering to apodemal vinculum. The posterior face of saccus seems more membranous with deep central invagination (Figure 8c).

Short straight dorso posterior margin and disto posterior (minutely toothed) margin blend into one continuous shallow convex curve producing posterior corner. Disto posterior process plunges ventrally producing, deep, broad, round ended process, with near parallel posterior and anterior margins (Figure 8a). Ventro posterior margin complex sinuous, folding laterally and dorso-ventrally, which along with intricately folded pseudotegumenal plate that folds inwards towards midline, produces a most remarkable sinuous folded effect (Figure 8b). Pseudotegumenal arms scoop-like due to plates curved inwardly (Figure 8b,c). Arms very short, broad, round ended; effect enhanced by significant broad convex ridge that runs from latero-posterior corners of pseudotegumenal rim directly towards midline then curves abruptly to run anteriorly. This has two effects, it makes the posterior of pseudotegumen very broad but depresses the plate anteriorly, shortening it along anteroposterior line. Basal rim wide but very short laterally. Twin processes substantial, triangular with posterior lean.

Valva have substantial square sacculus (Figure 8b) with distal arms arising from latero-ventral corner. Distal arms develop from prominent rounded ridge on sacculus face, producing distal arms with an inward leaning, flattened, paddle-like end. Distal arms and ridges densely covered in long setae.

Trulleum with four diamond shaped corners, juxta more molar like (Figure 8b).

***Remarks***

Grey and white moth with a remarkable and striking, pseudotegumen unlike any other species in this genus. Only two specimens were collected of this species. The second, Paratype, is smaller, forewing 30 mm, hindwing 25 mm. No female has been collected for this species.

***Distribution***

Collected from the Geraldton Sandplains biogeographic region in Kalbarri National Park on 5 June 2023, in semi-arid, low density, low height woodland (Figure 1).

***Etymology***

The dramatic and unusually curved ventral margins of pseudotegumen needs to be highlighted. Irregular and looking more like deep coastal bays (fjords) we landed upon a Greek origin word, kolpodes, which seemed to suggest more irregularity than the Latin, sinus or English sinuous.

#### 3.2.3. ***Abantiades profundus*** **sp. nov.** (Figure 9, Figure 10 and Figure 11)

urn:lsid:zoobank.org:act:24186D67-BB77-46F3-B84C-724049AF8A2F

***Material Examined***
***Holotype***
  In WAM. ♂. West. Aust. Geraldton Sandplains | Kalbarri National Park | Kalbarri, 27.7164° S 114.3244° E | 5 June 2023; M&M Moore || Spec. No. | 23184 | Leg removed | For tissue | storage | M.D. Moore.
***Paratypes***
  In WAM. ♂. West. Aust. Geraldton Sandplains | Kalbarri National Park Kalbarri, 27.7164° S 114.3244° E | 5 June 2023; M&M Moore || Spec. No. | 23180 | Leg removed | For tissue | storage | M.D. Moore.
  In SAMA. ♂. West. Aust. Geraldton Sandplains | Kalbarri National Park | Kalbarri, 27.7164° S 114.3244° E | 5 June 2023; M&M Moore || Spec. No. | 23183 | Leg removed | For tissue | storage | M.D. Moore ||| SAMA No. 31-21860.
  In SAMA. ♂. West. Aust. Geraldton Sandplains | Kalbarri National Park | Kalbarri, 27.7164° S 114.3244° E | 5 June 2023; M&M Moore || Spec. No. | 23179 | Leg removed | For tissue | storage | M.D. Moore ||| SAMA No. 31-21861.

***Diagnosis***

A striking moth with bright pinkish chestnut forewing and head, whitish pink hindwings and abdomen and yellow antennae.

The bright chestnut colouring of the males are only likely to be confused with *A. paradoxa*. They can be distinguished from that species by their ramal shape. In *A. paradoxa* rami distinctly bi-forked and “Y” shaped, whereas in *A. profundus* **sp. nov.** rami are un-forked and shaped like an inverted column with its base set distally. The genitalia shape differs too, being overall more triangular in *A. paradoxa*, more rectangular in *A. profundus* **sp. nov.** *Abantiades paradoxa* also has a distinct and obvious formed ridge on the side of the pseudotegumen, absent in *A. profundus* **sp. nov.** (Figure 11a). Twin processes also differ being triangular in *A. paradoxa* and cylindrical in *A. profundus* **sp. nov.**

The mtDNA COI ‘barcode’ sequences are available from GenBank (https://www.ncbi.nlm.nih.gov/genbank/; lodged 5 March 2026) for the paratypes B033 (23180; PZ098956), B036 (23183; PZ098957) and B032 (23179; PZ098955).

**Figure 9:**
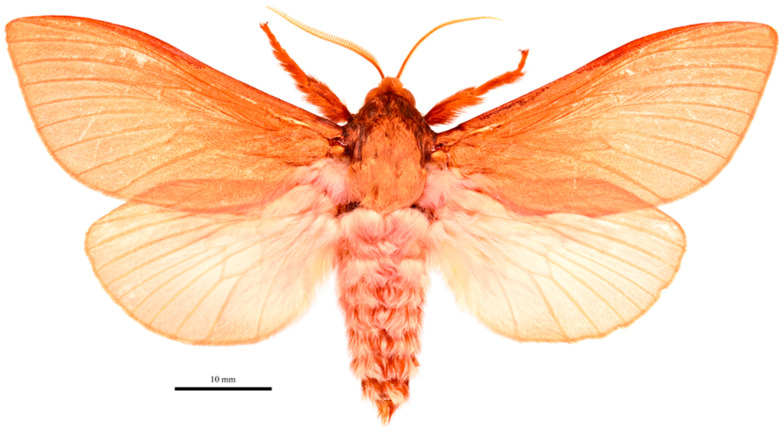
*Abantiades profundus* **sp. nov.**, habitus of male holotype, dorsal view. Geraldton Sandplains | Kalbarri National Park | Kalbarri. 27.7164° S, 114.3244° E | 5 June 2023; M&M Moore.

***Description***

***Male*** (Figure 9)

*Head*

Two large compound eyes dominate the head capsule.

**Figure 10:**
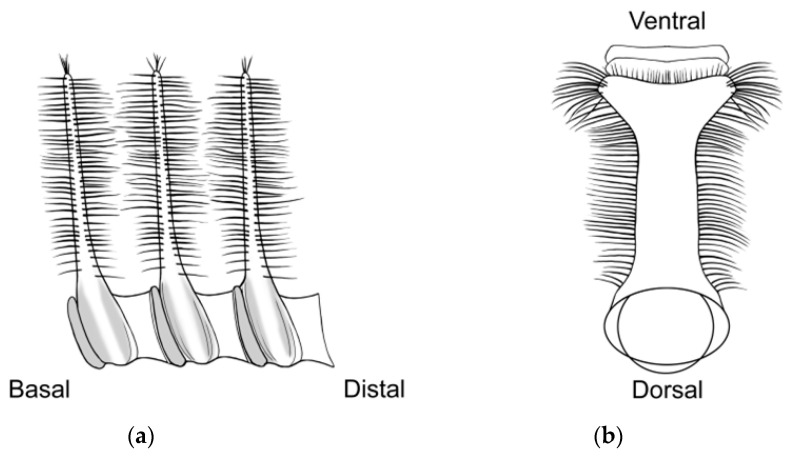
Antennal rami of male *Abantiades profundus* **sp. nov.**: (**a**) lateral view of rami, and (**b**) looking distally, rami in basal half of flagellum.

Antennae (Figure 10): 62 segments, one-third the length of the costa. Flagellum brown-yellow, downward projecting unipectinate stems white yellow, un-forked truncated ends bright yellow. Rami change shape along flagellum, majority wider and have a truncated flattish margin distally. Broad, obtuse points are produced at the corners and are densely planted with long colourless setae; distal margin is gently sinuous with shallow central concavity, supplanted, moderate length colourless setae along entire length. Colourless setae continue down the outer margins of the stems onto the segment bases. Faces of stems flat, sparsely covered in very short setae.

Labial palps: project slightly downward, three segmented densely covered in chestnut linear scales. Two basal segments almost co-equal (mid slightly longer), cylindrical with enlarged distal ends; distal segment smallest, spherical. Fronto-clypeus densely covered in erect, linear chestnut scales. Crown and nape, similar scales but light brown.

*Thorax*

All densely covered in piliform scales. Dorsal: Pro-thorax and meso-thorax covered light brown scales, meta-thorax covered, white scales suffused pink. Ventral: Scales, light buff suffused pink.

Forewing, 40 mm. Dorsal: Plain and unpatterned, membrane whitish transparent, veins yellowish white. Costa, densely covered in short, lanceolate, red chestnut scales. Area C to Sc light brown, densely packed with spathulate scales, variable length, longer at wing base, shorter apically. R vein covered red chestnut scales. Outer expanse wing, minimally covered in obtuse ended, leaf shaped, chestnut, scales. Distinct, patch of brown tipped whitish linear scales in wing base. Basal dorsum area densely covered, white, piliform, scales suffused pink. Fringe scales from apex to CuA1 golden pink square scales, thereafter white/pink linear. Ventral: Costa as above. C to Sc densely packed in whitish coloured scales, long linear at wing base to short lanceolate apically. Terminal one-third of apical area minimally covered in pink lanceolate scales, inner two thirds wing sparsely covered in whitish or pinkish piliform scales. Intensity of pink diminishes towards dorsum but wing basal areas intensely pink. Veins highlighted in pink or white scales.

Hindwing, 30 mm. Dorsal: Plain and unpatterned; all sparsely covered in white piliform scales suffused pink; wing base densely packed with pinkish piliform scales. Apex and wing margin fringe, browner, square cells to CuA1 thereafter linear. Ventral: Pinkish, similar to forewing though greater area piliform covered. Costa densely clothed, buff coloured, linear, scales. Veins highlighted with pink or white scales.

Legs. All legs densely scaled; dorsally and laterally longish linear, femur light brown, rest, bright red chestnut; ventrally, femur, tibia straw, tarsi, short, fine, golden-straw-coloured scales. Epiphysis present inner tibia, large vase shaped with point on distal margin covered in linear scales.

Arolium tear drop shaped, point distal.

*Abdomen*

Dorsal: densely covered in long white or pink, piliform scales. Ventrally, buff suffused red long piliform scales.

**Figure 11:**
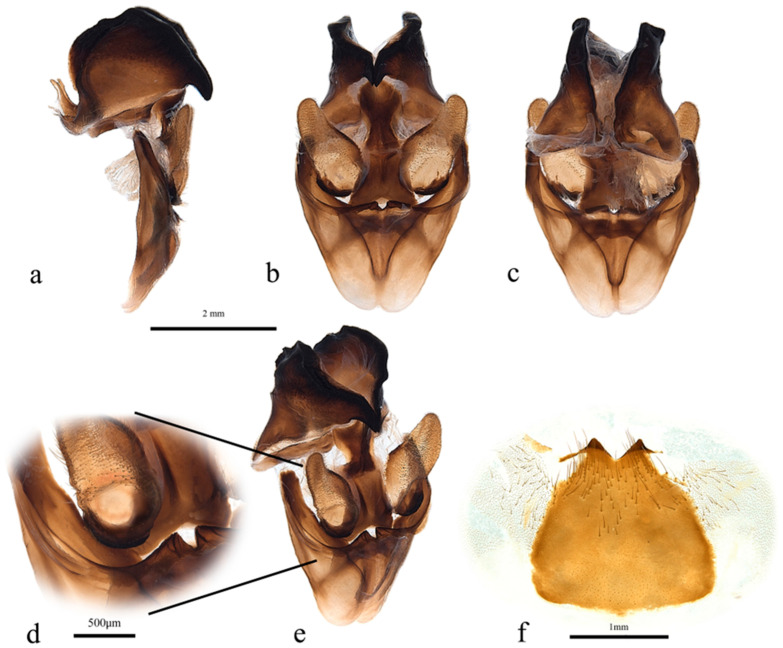
*Abantiades profundus* **sp. nov.**, male genitalia ((**a**–**e**): ventral top; dorsal bottom) (**a**) lateral, (**b**) anterior, (**c**) posterior, (**d**) enlarged image of valva showing depression in sacculus, (**e**) anterio-lateral, and (**f**) sternite 8 (posterior top; anterior bottom).

Sternite 8 (see Figure 11f). Anterior margin broad convex meeting inward angling lateral margins in broad obtuse rounded corners. Shallow convex sides, distally, angle more sharply towards medial line, meet short, strongly angled, posterior margin in two strongly sclerotised points that have short lateral extensions and produce a strongly angled deep sided cleft medially. The points on dorsal margin are coherent with the saccus ventral margin making physical separation difficult.

Genitalia (Figure 11a–e). Vincular arms are somewhat straight attached broadly but slightly below their apices, producing a narrower join. Proximally broad they continually narrow, in distal third start to twist and curve back towards midline; end, cut square. Produce deep, narrow, ‘V’ shaped saccus with obtuse curved apex. Ventral margin of saccus, broad, strongly sclerotised, centrally convex curved with deep central indentation (Figure 11b,c). Lateral arms of saccus long, sclerotised, start broad then narrow and attach to vincular arms twisting distally.

Pseudotegumen, heavily sclerotised, rectangular shape (slightly rotated), dorso-posterior margin long and straight producing a broad convex posterior corner set low on pseudotegumen. Disto-posterior margin slightly sinuous meeting small triangular convex curved disto-posterior projection in slight concavity (Figure 11a). Ventro-posterior margin, short, straight meeting relatively long, straight ventral margin of pseudoteguminal arms in concave bump. Pseudoteguminal arms relatively short and straight with curved anterior and pointed apex. Basal rim projects posteriorly merging into two short broadly based cylindrical twin processes that are chitinous at the base but membranous distally. These processes fail substantially to reach posterior corner.

Intermediate plate, substantial, trapezoidal in shape and closely apprised to pseudotegumen (Figure 11a).

Valva oblate, sacculus broad roundish, with dorsal, elliptical, setose free, depression bordered dorsally by a heavily sclerotised ledge. Distal arms broadly join sacculus along entire width and remain broad to their rounded, obtuse apex (Figure 11d,e). Apart from depression all amply covered in mid length setae.

Trulleum longish shaped like inverted chalice, juxta large molar shape, both structures heavily sclerotised (Figure 11b).

***Remarks***

Four males were collected varying only slightly in size from 34 mm to 40 mm forewing length. We also note that many *Abantiades* on emergence are coated with a coloured suffusion. In this species it is reddish pink, in *A. paradoxa* it can be magenta to bright purple. Unfortunately, this colouring fades quickly leaving the moth with a much less vibrant appearance.

***Distribution***

All specimens were collected in the one location in Kalbarri National Park, in low height, low density woodland occupying a ridge line on sand dunes to the south of Murchison River on 5 June 2023 (Table S1).

***Etymology***

The original member of the clade was named paradoxa by Tindale (1932) because, we believe, compared to all other *Abantiades* species known at the time the moth, with its plain, unpatterned chestnut colouring was superficially different and distinctive. This new species, though similar to *A. paradoxa*, is quite spectacular with profoundly different antennae, hence the name profundus.

#### 3.2.4. ***Abantiades lepusaures*** **sp. nov.** (Figure 12, Figure 13, Figure 14, Figure 15, Figure 16 and Figure 17)

urn:lsid:zoobank.org:act:76E813C6-5F5F-4ED8-A9D8-30148D22EE68

***Material Examined***
***Holotype***
  In ANIC: ♂. 31°11′36.5″ S 120°32′47.8″ E | AUSTRALIA, WA, 70 km W | Coolgardie, Goldfields | Woodlands NP. 17 April 2007 | MV-lamp & UV-flt. | A. Zwick and G. Cocking.
***Paratype***
  In ANIC: ♀. 31°11′36.5″ S 120°32′47.8″ E | AUSTRALIA, WA, 70 km W | Coolgardie, Goldfields | Woodlands NP. 17 April 2007 | MV-lamp & UV-flt. | A. Zwick and G. Cocking.
***Other material examined***
  In ANIC: ♀. [No label with specimen. Likely to have been collected with the holotype and paratype above.]

***Diagnosis***

**Figure 12:**
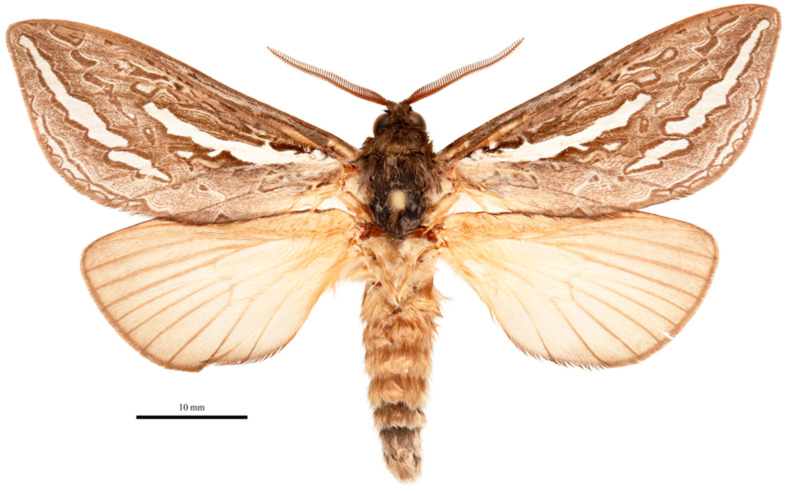
*Abantiades lepusaures* **sp. nov.**, habitus of male holotype, dorsal view. 31°11′36.5″ S 120°32′47.8″ E | AUSTRALIA, WA, 70 km W | Coolgardie, Goldfields | Woodlands NP. 17 April 2007 | MV-lamp & UV-flt. | A. Zwick and G. Cocking.

***Male*** (Figure 12)

A smaller *Abantiades* species possessing a highly patterned brown forewing with two large, discrete, silvery white marks, a curved buff spotted line in the tornal region and a post medial interrupted brown line. Hindwing unpatterned, whitish grey with veins defined. Dark grey head thorax, abdomen light grey.

Possible confusion with *A. albofasciatus*, *A. neglecta*, or *A. pica*, as all have light coloured hindwings and brown forewings. Antennal morphology will separate *A. albofasciatus* and *A. neglecta* because they have un-forked rami whereas *A. lepusaures* **sp. nov.** is bi-forked. *Abantiades pica* is also bi-forked but forks are filamentous and linear, with a distinct “U” at the minute pectinate stem. *Abantiades lepusaures* **sp. nov.** however has broad forks, not filamentous, erupting in a “V” form from a longer stem. Wing pattern and colouration is also different in *A. albofasciatus*, having a greyish forewing with only one long white line on forewing, stretching from base to near apex, with a grey-white hindwing. *Abantiades neglecta* has a dark coloured forewing with two large white marks with white hindwings, but *A. pica* is more similarly patterned and coloured to the new species. *Abantiades pica*, however, is a smaller moth with a discoidal white line that is crescentic in shape and almost reaches the costa. In *A. lepusaures* **sp. nov.** the discoidal mark is only slightly crescentic. *Abantiades pica* also has a significant posterior highlight to discoidal white mark and an obvious dark brown spot with white centre close to the wing axil. In *A. lepusaures* **sp. nov.** the highlight is much reduced and has no brown spot.

**Figure 13:**
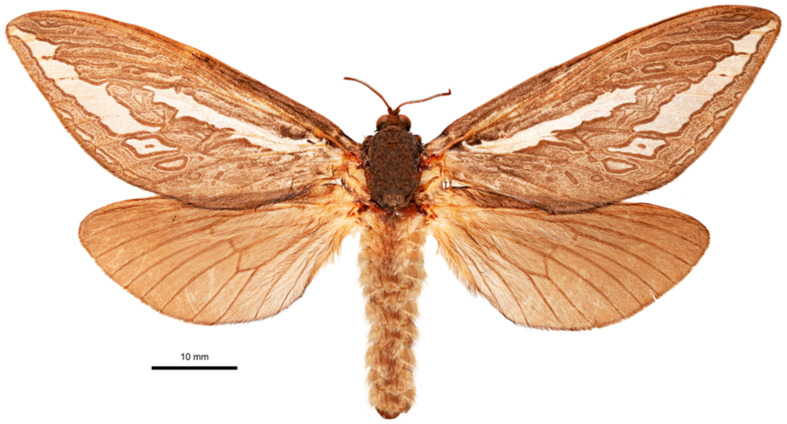
*Abantiades lepusaures* **sp. nov.**, habitus of female paratype, dorsal view. 31°11′36.5″ S 120°32′47.8″ E | AUSTRALIA, WA, 70 km W | Coolgardie, Goldfields | Woodlands NP. 17 April 2007 | MV-lamp & UV-flt. | A. Zwick and G. Cocking.

***Female*** (Figure 13)

Plain, medium brown coloured moth with two large white marks and third smaller line in tornal/terminal area. Subterminal mark broader than usual. Forewing heavily scrolled, hindwing unmarked, yellower than forewing. Bi-forked antennae and wings of narrower form.

Among bi-forked *Abantiades* the lanceolate shape to the tines of *A. lepusaures* **sp. nov.** will be a point of separation; *A. paradoxa*, and *A. concordia* females have tines that are linear cylindrical in form and *A. karnka* and *A. pica* have tines that are thin filamentous.

The genitalia of *A. lepusaures* **sp. nov.** is also different in that the triangular lobes on the dorsal plate are unusual. *Abantiades paradoxa* has more normal, rounded lobes (and a large area of dense setae on the lateral plates of the antevaginal lamellae). Both *A. lepusaures* **sp. nov.** and *A. pica* have similar antevaginal lamellae, however, these two species can be separated on their differing medial lobes. In *A. pica* it is of triangular form with a deeply invaginated inner margin and insubstantial lateral wings, whereas in *A. lepusaures* it is rectangular with substantial lateral wings.

Forewings of *A. lepusaures* **sp. nov.** are mid-brown with two distinct white marks, whereas *A. karnka* is black in colour whilst *A. paradoxa* and *A. concordia* are orange/chestnut in colour and have no white marks. Forewings of *A. pica* are similar to *A. lepusaures* **sp. nov.**, however *A. pica* has a discoidal mark that is crescentic (as in the male) and a well-defined post medial line of smaller spots.

The mtDNA COI ‘barcode’ sequences are available from GenBank (https://www.ncbi.nlm.nih.gov/genbank/; lodged 5 March 2026) for the paratype male (F020; PZ098959) and the paratype female (D003; PZ098958).

***Description***

***Male***

*Head*

Two large compound eyes dominate head capsule. Fronto-clypeus densely covered with upright, light grey, blunt ended, linear, scales. Crown densely covered in upright cinerous-grey scales.

**Figure 14:**
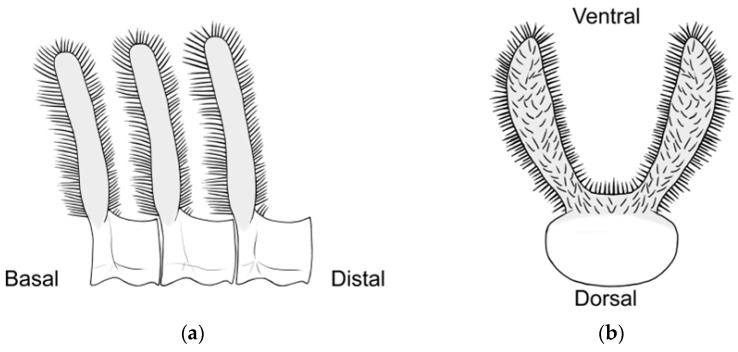
Antennal rami of male *Abantiades lepusaures* **sp. nov.** (**a**) Lateral view of rami, and (**b**) looking distally, rami in basal half of flagellum.

Antennae (Figure 14): 56 segments, 40–45% length of costa. Flagellum yellow-brown basally, more reddish distally. Rami light reddish-brown, marginally unipectinate, stem very short projecting laterally producing two forks, whose inner margin is straightish, outer margin convex. Rami faces flat. Basal rami bi-lobed, whereas distal rami bi-forked, smaller but well formed. All densely covered in short, colourless, setae.

Labial palps: Three segmented, basal segment cylindrical, distal end broader, mid segment longest, cylindrical, distal segment smallest, spherical. Whole clothed in fine linear scales; light grey around basal segment graduating to a very dark grey on distal segment.

*Thorax*

Dorsal: Pro and meso segments densely covered cinereous grey, piliform scales, meta longer piliform scales, white. Ventral and lateral: piliform scales, mix pale straw, mid grey.

Forewing, 33 mm. Dorsal: Costa densely covered in short, blunt ended, leaf shaped, dark brown scales, paler apically. C to Sc space filled with mixture of brown/white/grey, fine, linear, scales. Two large white marks. One subterminal, from just short apex to CuA2; lens shaped but interrupted; filled, shining white scales, narrowly bordered dark brown, itself narrowly bordered by buff scales. Termen side filled with mixture of buff and brown scales until reaches a scalloped, curved, buff/white line, bordered dark brown stretching from Rs4 to Anal vein. Discoidal white, slightly crescentic, irregular shape, filled shining white scales bordered dark brown which widens to the anterior and posterior producing highlight areas. Anterior to this, long buff/dark brown scroll lines plus other brown and white marks. Posterior to discoidal line buff areas with occasional white or brown marks. Between two larger markings an irregular, interrupted brown line edged buff. Wing fringed red-brown. Ventral: Costa densely packed with short blunt ended brown, scales, slightly lighter distally, darker more piliform basally. Costa to Sc space brown scales longer towards Sc. Dorsal wing marking visible, Termen, sub-termen and apical areas sparsely covered in light cinnamon brown, long, leaf-like scales. Basal and posterior areas, scales sparser, piliform. White coloured areas centrally, straw elsewhere.

Hindwing, 24 mm. Dorsal: Membrane whitish. Wing unpatterned, veins highlighted gold straw. Costa densely covered dark brown scales, rest wing very sparsely covered, long linear scales, piliform basally. Forward Rs4 golden straw, posterior white. Ventral: Costa densely covered golden straw, short piliform, scales. C to Sc space golden straw scales broader and shorter apically, longer piliform medially, white piliform basally. Termen, sub-termen and apical areas linear scales. Anterior of M1, light cinnamon brown, posterior M1 to CuA2 white, posterior CuA2 pale straw. Scales more piliform towards wing base.

Legs. Dorsal and lateral dark cinerous grey, inner light straw. Arolium tear drop shaped, point distal.

*Abdomen*

Densely covered in long piliform scales. Dorsal: Tergites 1–5 white. 6–7 grey, tipped white distally. Ventral: Straw coloured. Lighter proximally darker distally.

**Figure 15:**
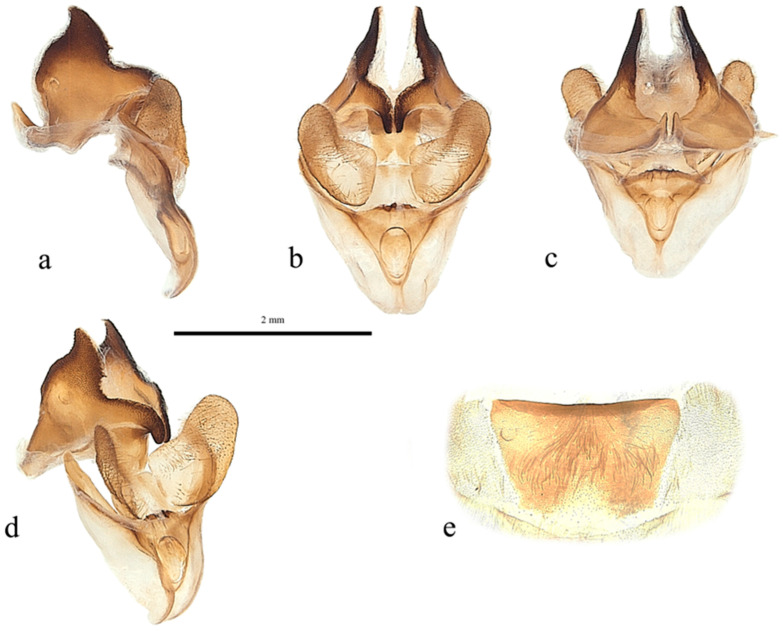
*Abantiades lepusaures* **sp. nov.**, male genitalia ((**a**–**d**): ventral top; dorsal bottom): (**a**) lateral, (**b**) anterior, (**c**) posterior, (**d**) antero-lateral, and (**e**) sternite 8 (posterior top; anterior bottom).

Sternite 8 (Figure 15e). Posterior margin slightly concave, lightly reinforced with extra sclerotisation. Sides, posterior convex curved, basally minutely concave, angles towards midline. Anterior margin bilobed with deep, central, diffusely defined, invagination.

Genitalia (Figure 15a–d). Vincular arms join broadly, retaining width throughout apodermal length. Relatively straight inner margins produce a regular ‘V’-shaped, pointed, blunt ended saccus. The anterior face of the saccus lightly sclerotised and has a convex shaped ventral margin which is more heavily sclerotised and minutely toothed centrally (Figure 15b). Ventral corners of triangular faced saccus extend laterally and adhere strongly to vincular arms, crossing over arms and up sides. Distal vincular arms bend on long axis, distal portions flat, narrow, and thin. Posterior face of saccus, membranous with deeply concave, ventral margin (Figure 15c).

Pseudotegumen, complex shape; heavily sclerotised along external margin particularly disto-posterior projection (Figure 15a). Dorso-posterior margin relatively long, concave, disto-posterior corner somewhat prominent. Disto-posterior margin continues convex margin of disto-posterior corner to the backward leaning apex of triangular disto-posterior projection. Continuous convex anterior margin of disto-posterior projection merges in a short sinuous ventro-posterior margin, which itself produces a noticeable concavity where it meets the ventral pseudotegumenal arms (Figure 15a). The dorso-anterior margin of the pseudotegumen, project the arms well forward before they turn dorsally producing short, rounded ends (Figure 15a).

Lateral expansion of pseudotegumen produces posterior set, wide wing like lateral expansions of the basal rim. Attached to dorso-anterior margin of the pseudotegumen is intermedial plate, which in this species looks parallelogram in shape. Posteriorly the basal rim angles strongly to the vertical were chitinous, sclerotised, triangular twin appendages project, failing to make the disto-posterior corner.

Valvae (Figure 15b,d) slab like, have a large square sacculus and distal arms that attach to the sacculus along its entire width, broad blunt apices. Slight central ridge in face bends distal arms inward slightly. Distal arms and central ridge in sacculus populated with long setae.

**Figure 16:**
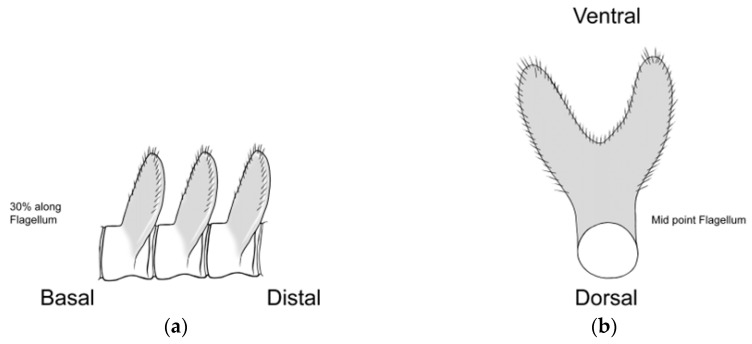
Antennal rami of female *Abantiades lepusaures* **sp. nov.** (**a**) Lateral view of rami, and (**b**) looking distally, rami in basal half of flagellum.

***Female*** (Figure 16)

*Head*

Densely clothed in brown, blunt ended, linear scales. Two large compound eyes dominate the head capsule.

Labial palps 3 segmented: basal vase shape; middle, longest, cylindrical, linear; distal, smallest, sub spherical.

**Figure 17:**
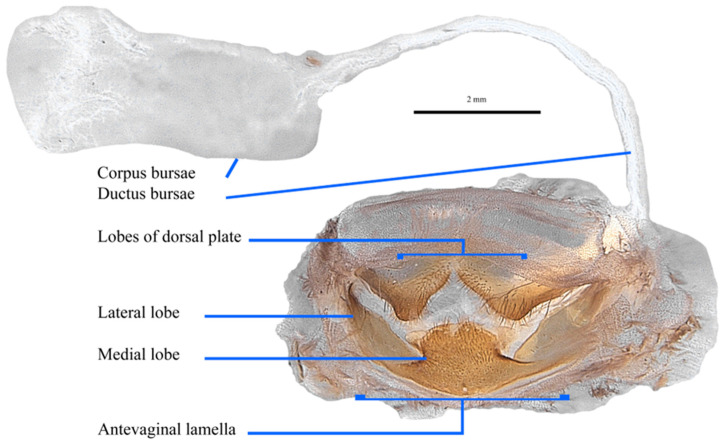
*Abantiades lepusaures* **sp. nov.**, female genitalia. Posterior view of genitalia (dorsal top; ventral bottom) showing dorsal plate and antevaginal lamella, lateral plate of antevaginal lamella, and the ductus bursae and corpus bursae.

Antennae (Figure 17): about one seventh length costa; 61 segments, bi-forked rami. Flagellum yellowish; rami red-brown, tines darker. Rami project ventrally; stem as broad as flagellum, tines have wide face, approximately twice stem height but shape and proportions vary along flagellum; first seven and last three flagellum segments un-forked. Anterior and posterior tines similar in shape, though posterior slightly rotated making it look slightly thinner; lateral margins continually curved, internal margins much less so, hence tines wider nearer apex. Setae sparse, most numerous along margins; longest on tine apex.

*Thorax*

Densely clothed in piliform scales; ventral, brown; dorsal and lateral, yellow-straw.

Forewing: 50 mm. Veins yellowish, membrane white but translucent. Dorsal: highly patterned; liberally covered in buff and brown scrolling lines and shapes; lines in marginal regions, shapes in apical and central areas; dorsal areas less patterned: Costa densely clothed in fine, short, blunt ended, golden-straw coloured, linear scales; C to Sc space densely clothed in similarly shaped though larger scales; some spathulate. Rest wing well covered in mixture of longish, acute and obtuse tipped scales. Fringing scales, spathulate, apex to CuA2, thereafter short piliform. Two large prominent white marks, both filled with glossy cream-white scales and a thin dark brown border. Discoidal; linear, about 60% wing length. Dark brown scales to anterior and posterior in basal area provide marginal highlighting. Subterminal; longer and broader, from apex to 1A; disjointed at anal end. Also, narrow, terminal line of disjointed, buff coloured, rectangular, marks in tornal region. Ventral: Unpatterned, light brown; dorsal markings visible. Scales long linear near margins lengthening towards base; become more sparse and piliform.

Hindwing: 40 mm. Dorsal; Plain, unpatterned, yellowish-brown; straw coloured wing base. Ventral; unpatterned; scales similar to forewing though more linear with yellowish tinge.

Legs: cinerous brown dorsally, yellow-straw ventrally. Arolium elliptical.

*Abdomen*

Dorsal/Ventral similar; anterior straw yellow, posterior brown.

**Figure 18:**
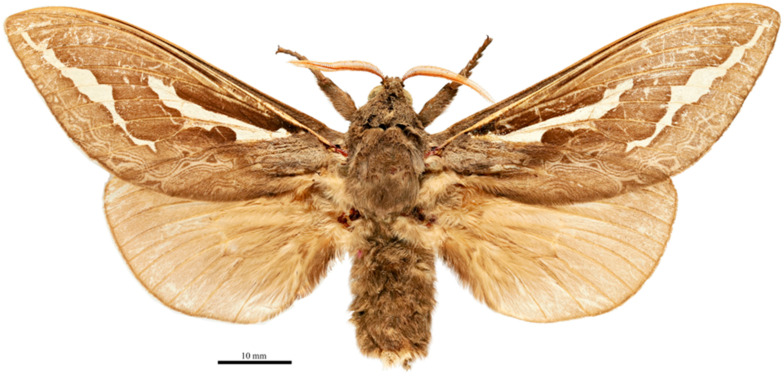
*Abantiades incognito* **sp. nov.**, habitus of male holotype, dorsal view. Toowoomba | 10.4.43 || Spec. No | 21062 | Leg removed | for tissue | storage | MD Moore ||| Presented by | E.J. Dumigan | 1966 |||| 32060 ||||| QM Reg. No. | T234262.

Genitalia (Figure 18). Dorsal plate bilobed each lobe triangular with concave curved sides producing thick blunt ended points that are well covered in longish setae. Numerous setae also line central invagination and area behind. Some sparse but longish setae on lateral margins. Antevaginal lamellae trilobed; medial plate broad based, projecting inwardly, rectangular base, round topped; distal end somewhat membranous with lateral wing like projections. Lateral plates flattish, less sclerotised, virtually setae free.

***Variation***

Second specimen smaller, forewing 40 mm, hindwing 30 mm. Subterminal mark not disjointed. Terminal mark more substantive and extends further along termen.

***Remarks***

This new species was part of fieldwork in Western Australia in 2007 in the Eastern Goldfields area that also resulted in a now described species *A. malleus* Moore and Beaver, 2022. Those collections now yielding a second new species described here as *A. lepusaures* **sp. nov.** and highlights the need for more collecting in the regional areas of WA.

The second female specimen has not been made a paratype because it does not have any data attached.

***Distribution***

Coolgardie, Woodlands National Park, WA (Table S1).

***Etymology***

In the male particularly the shape of the ramal forking reminded us of the shape used for toy or cartoon rabbit ears, hence lepusaures. Latin for rabbit ears.

#### 3.2.5. ***Abantiades incognito*** **sp. nov.** (Figure 18, Figure 19 and Figure 20)

urn:lsid:zoobank.org:act:B2C5CC90-DA5B-448D-9109-0106F154848B

***Material Examined***
***Holotype***
  In QM. ♂. Toowoomba | 10.4.43 || Spec. No | 21062 | Leg removed | for tissue | storage | MD Moore ||| Presented by | E.J. Dumigan | 1966 |||| 32060 |||||QM Reg. No. | T234262.
***Paratypes***
  In QM. ♂. Mt. Glorious | S.E.Q. | A. Hillier | 9 v 1972 ||.
  In QM. ♂. Lam. Nat. Pk. Qld. | 19 – 22 May 1963 | J.C. Cardale || QM Reg. No. | T234249 ||| 4.

***Diagnosis***

This is a large moth, similar to *A. atripalpis* in external appearance, being dark with little scrolling on wings and two large irregular linear white marks on forewings, the discoidal mark being highlighted to the anterior and posterior by dark brown scales. However, *A. atripalpis* has three distinctive features, absent in this new species: they are, pseudotegumenal arms end in a distinct knob; large chitinous tag on vincular arms; sternite 8, folded antero-posteriorly, on itself with a large central depression (for comparison with *A. atripalpis* genitalia, compare Figure 2 with Figure 20a,b). *Abantiades argentata*, has similar morphology in sternite 8 and general structure of saccus but differs from *A. incognito* **sp. nov.** in shape of pseudotegumen. In *A. argentata* the pseudotegumen is distinctly and obviously triangular in form, with a pronounced, larger and more rounded set of pseudotegumenal arms. In *A. incognito* **sp. nov.** the pseudotegumen is not overtly triangular but much more rounded with shorter, neater, more compact pseudotegumenal arms, set somewhat underneath the pseudotegumen (Figure 20a) rather than in the front of it. The form of the genitalia is very similar between *A. inexpecta* and *A. incognito* **sp. nov.**, except *A. inexpecta* has narrow, cylindrical, straight, twin processes, and in *A. incognito* **sp. nov.** they are cylindrical, thicker and curved; *A. inexpecta* has thicker, blunt ended pseudotegumenal arms whereas they are thinner and more pointed in *A. incognito* **sp. nov.** Antennal colour might also differ, a fresh specimen of *A. inexpecta* has black antennae, whereas antenna are russet in the older *A. incognito* **sp. nov.** specimen.

***Description***

***Male*** (Figure 18)

*Head*

Fronto-clypeus densely covered in erect, grey-brown pointed scales. Vertex; browner, densely covered in stiff, filiform scales, more hairlike towards rear. Eyes; large, black, compound just smaller than head capsule.

**Figure 19:**
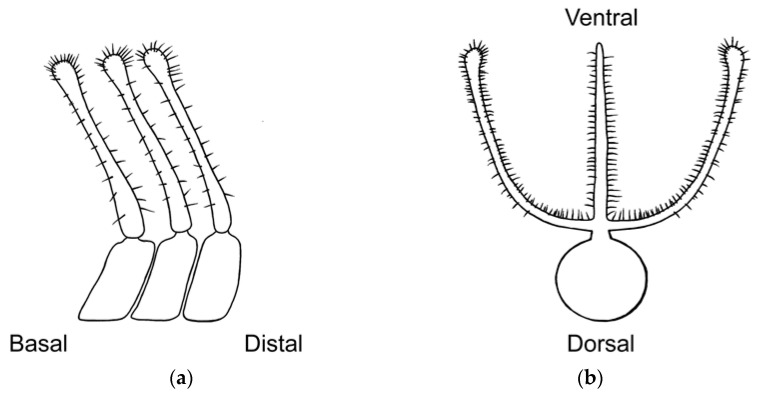
Antennal rami of male *Abantiades incognito* **sp. nov.** (**a**) Lateral view of rami rami, and (**b**) looking distally, rami in basal half of flagellum.

Antennae (Figure 19): Yellow, one third to one quarter length costa, 74 segments, projecting ventrally, with a stem about one-seventh length of ramus, broad faced, then stem splitting into three forks. Outer, project laterally then curve to project ventrally and distally, distal end flat, angled, over 10× longer than wide, inner surface covered in colourless setae about as long as fork width that project inwardly. Central fork, 7× longer than wide, sub circular, erect, projects ventrally to same level as outer forks, less densely covered, in colourless setae.

Labial palps: three segmented. First, cup shaped, distal margin angled; second, 1.5× the length first, approximately cylindrical, laterally bent towards distal end; third smallest, orbicular; all covered in stiff, prostrate, pencil like, yellow brown scales darker at ends.

*Thorax*

All densely covered in grey brown piliform scales, base lighter.

Forewing, 53 mm. Membranes white opaque, veins golden brown-yellow.

Dorsal: Costa, densely covered in small, lanceolate, golden-brown scales. Costa to Sc darker, densely covered in blunt ended, grey-brown scales, shorter apically, longer basally. Majority wing covered in relatively short, obtuse curve ended, leaf shaped scales; change as move towards base, become grey-brown, blunt ended, strap like, dorsally become piliform and blonde. Wing scales light warm brown. Two major white marks. Subterminal shining white, irregular shape, width, disjointed; from near apex, fails to make CuA1. Discoidal line, same colour, irregular shape and width, closely approaches subterminal line. Apically and dorsally, lines, swirls and circles of grey scales produce a mild scrolling effect.

Ventral: Upper white marks, show through. Sparsely covered in blonde and light brown scales, lanceolate in apical area, longer, denser, more piliform towards base.

Hindwing, 36 mm. Dorsal: Plain, lighter than forewing, brown-yellow. Sparsely covered in light brown filiform scales becoming piliform towards base. Ventrally: As forewing, scales more filiform distally.

Legs. Fore and mid-legs dorsally dark brown, with longer scales laterally, tarsomeres lighter. Ventrally femur golden, tibia mid brown, tarsomeres more yellow than tibia. Hindlegs dorsally, femur buff straw, tibia and tarsomeres mid brown, ventrally all lighter.

*Abdomen*

Darker than thorax, densely covered in piliform scales light grey base, grey-brown distally.

**Figure 20:**
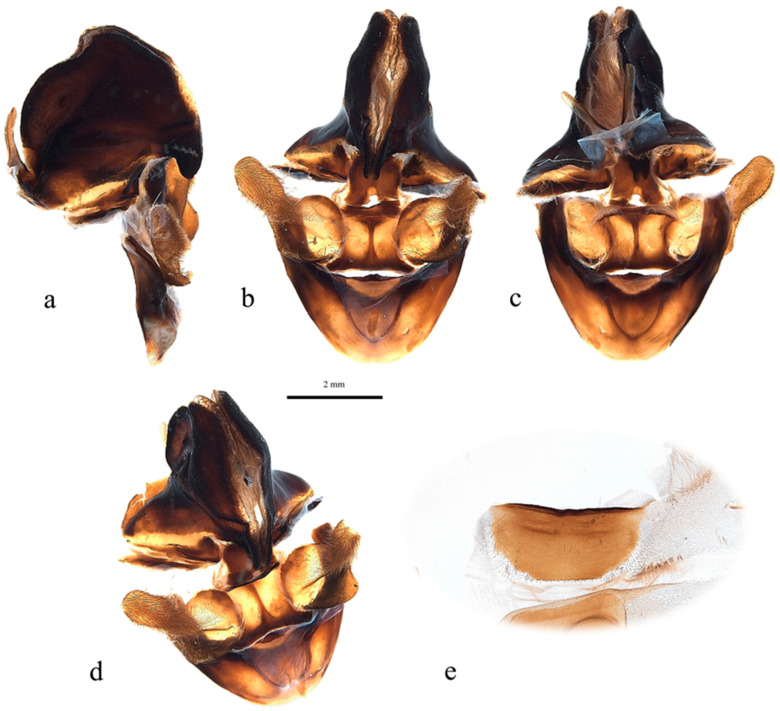
*Abantiades incognito* **sp. nov.**, male genitalia ((**a**–**d**): ventral top; dorsal bottom): (**a**) lateral, (**b**) anterior, (**c**) posterior, (**d**) antero-lateral, and (**e**) sternite 8 (posterior top; anterior bottom).

Sternite 8 (Figure 20e). Posterior margin distinctly sclerotised, slight folding, gently concave with small central prominence. Anterior corners acutely pointed, lateral margins convex curved towards medial line, anterior margin straight.

Genitalia (Figure 20a–d). Vincular arms broadly joined at proximal end. The arms increase in width reaching a maximum about one-third along length whence they narrow over distance to generate short, squat, heavily sclerotised, distal arms that have truncated ends. The saccus generated is somewhat ‘V’ shaped with dorsal apex obtuse curved. Anterior face sclerotised, ventral margin straightish, with raised central prominence. Dorsal face deeply concave ventral margin (Figure 20c).

Pseudotegumen heavily sclerotised, and except for triangular disto-posterior projection, demonstrates a general roundness to structure. Dorso-posterior margin short, straight. Broad, rounded, convex posterior corner. Size posterior corner leaves room for only a short straight disto-posterior margin before becoming part of distinctly triangular dorso-posterior projection with short steep posterior margin and longer shallower anterior one, giving impression of keel-like shape. The ventro-posterior margin is straight bent steeper about halfway along length, producing distinct shallow broad prominence. Ventro-posterior margin smoothly joins the ventral margin of the pseudotegumenal arms which dorsally curves more steeply producing a round obtuse angled apex. Margin falls steeply to meet broadly concave anterior margin of the pseudotegumen ending long and straight before meeting short basal rim proper. Pseudotegumenal arms appear short, compact.

Pseudotegumenal rim rounded, projects to posterior where turns ventrally to produce a substantial base for membranous cylindrical twin processes that almost reach apex of posterior corner (Figure 20a).

Valva (Figure 20b,d) have rounded sacculus. Distal arms equal width and length of sacculus. Ridge running down distal arm and sacculus set to anterior producing small, flattened area ventral to substantial depression in sacculus (Figure 20a,c). Sacculus has dorso-lateral projections.

***Remarks***

Lamington National Park specimen, forewing 62 mm, hindwing 42 mm. Mount Glorious specimen forewing 65 mm, hindwing 43 mm. The scrolling in the Mt. Glorious specimen is limited to a few lines in the dorsum area of the forewing, whilst the general shape of the pseudotegumen is consistent with other specimens but particulars of posterior corner, and disto-posterior projection varies as does the ventral margin of saccus.

Tendency of older specimens to lose their colour as they age makes description and comparison difficult. Older specimens resemble *A. atripalpis* and can only be distinguished by dissection.

*Abantiades incognito* **sp. nov.** genitalia has a likeness to *A. inexpecta*, a WA species recorded from the Coolgardie-Norseman-Balladonia area. The habitats though are very different with *A. inexpecta* living in semi-arid mallee conditions and *A. incognito* **sp. nov.** from the Great Dividing Range in SE Queensland and northern New South Wales in wet sclerophyll habitat.

***Distribution***

Three elevated locations of Toowoomba, Mount Glorious and Lamington National Park in SE Queensland (Table S1). As the SE Queensland biogeographic region goes into northern NSW the distribution of this species may extend to the south. Collection dates range from 10 April to 22 May.

***Etymology***

Incognito, means unknown or not examined in Latin; in English, hiding one’s identity. We think this appropriate because, it has not been previously examined, and it superficially resembles *A. atripalpis*.

## 4. Discussion

The diversity within the hepialid genus *Abantiades* increased markedly from 14 species [5] to 37 with the inclusion of the 14 new species and nine added from synonymising the ‘*Bordaia*’ and ‘*Trictena*’ genera into *Abantiades* [1,9,10]. A further 10 have also been described recently [8,11,12,13], increasing the number of species known from the genus *Abantiades* to 47 (see also [2]). With the five new species described here, we have now further increased the known species in the genus to 52 (Table 1). The genus *Abantiades* continues to grow as more targeted collections during adult emergence periods are undertaken in under-represented locations. There are many areas in Australia where little or no collections have occurred, and so the number of species in the genus *Abantiades* is likely to further increase, as is the case for all the hepialid genera in Australia.

Here, our new species, one with tri-forked rami, two with bi-forked rami, and two with un-forked rami, demonstrate problems with the use of the structure of the rami on the antenna. Only a single stem of the ramus erupts from each segment (of the antennal flagellum); therefore, all *Abantiades* species are unipectinate. In some species, the stem then forks once or twice to produce bi-forked or tri-forked rami, others do not fork and are termed un-forked (see Figure A2). Initially the amount of forking of the rami (wrongly termed ‘pectination’) was thought to be a taxonomic character defining the genus ‘*Trictena*’ by Meyrick [24] and later the genus ‘*Bordaia*’ by Tindale [5]; Simonsen [1] questioned this interpretation (but retained the terminology) and synonymised the two genera with *Abantiades*. The mtDNA *COI* ‘barcode’ sequences here build on the study of 34 *Abantiades* species [8] with all currently available species (49 of 52) within the genus *Abantiades*. Despite the caveats with mtDNA *COI* [33,34], it is clear that this character, involving the forking of the rami on the antennae, is paraphyletic with respect to all *Abantiades* species in the genus (Figure 4).

The *Abantiades* tri-forked species now include 18 species, with 13 known from, or extend into, the semi-arid environment. These tri-forked species are monophyletic but embedded within the genus *Abantiades*. Within this tri-forked clade, our new species *A. incognito* **sp. nov.** is closely allied, morphologically, with *A. inexpecta* in a subclade identified by Moore et al. [12] as the ‘short-beaked’ clade due to all members possessing short, straight, beak-like pseudoteguminal arms in the male genitalia, distinguishing them from other tri-forked *Abantiades* species that have longer, curved arms. Currently, there is no mtDNA *COI* evidence to unite the bi-forked species separately from the un-forked species (see Figure 2). The un-forked species are the most diverse, with 27 species (15 extending into the semi-arid environment), but are the dominate group in cold, temperate, and wetter regions in southern Australia. With the descriptions of our two new bi-forked *Abantiades* species, the number of bi-forked species increases to six. Five of these species are found only in Western Australia, all of which inhabit, at least partly, semi-arid zones (250 mm–350 mm rain per year). The sixth species, *A. pica*, also lives in semi-arid environments, living widely throughout the western Mallee, the Western Woodlands of WA and the eastern Mallee in SA and Vic. That it has been found on Kangaroo Island seems unusual [13].

While numerous diverse species have evolved with the semi-arid environment, the Geraldton Sandplains Region specifically reveals many unique species of plants [35], of which 51 are either considered critically endangered or endangered. In this region we have currently identified seven species of *Abantiades* moth. The three new species in this paper have currently only been found to inhabit the Geraldton Sandplains Region, with four species (*A. kayi*, *A. argentata*, *A. albofasciatus*, and *A. neglecta*) known to have more widespread ranges. *Abantiades kayi*, is a widely distributed species, but one that seems retained within the larger south-west Australia savanna ecoregion. *Abantiades argentata* is a species that may require *Eucalyptus camaldulensis*, of which there are only a few isolated stands in SW–Western Australia and along the central coast. This north-western population may be isolated geographically from the extensive populations in eastern Australia (see [11]). *Abantiades albofasciatus* is also known from the region but is currently found more widespread in WA. Although only known from WA, *A. neglecta* is the most widespread species and likely feeds on the widely distributed *Allocasuarina* species rather than the more usual *Eucalyptus*.

In addition to the seven *Abantiades* species from the Geraldton Sandplains Region above, seven additional hepialid species were collected or known from the region. We collected three species of *Fraus* Walker, 1856 (*F. pteromela* (Lower, 1892), *F. biloba* Nielsen and Kristensen, 1989 and *F. simulans* Walker, 1856. Two of the species (*F. pteromela* and *F. simulens*) have not been recorded as being that far north, but two other species have been (*F. biloba* and *F. basidispina* Nielsen and Kristensen, 1989). In the current information, it appears that most species of *Fraus* range widely and may not be as range-limited with regard to host plant specificity as other hepialids, but this requires further assessment and, in particular, greater sampling across Australia. Previous collections from this region also include *Aenetes djernaesae* Simonsen, 2018 and two species of *Oxycanus*. One *Oxycanus* sp. was collected at Cervantes; the second species was collected and identified by N. McFarlane in 1975 at Drummond Cove as *Oxycanus kochi* Tindale, 1955.

## 5. Conclusions

Our study has demonstrated historical confusion with the use of the structure of the rami as a character to delineate hepialid moths in the genus *Abantiades*. While the mtDNA *COI* currently groups the tri-forked species together, the un-forked and bi-forked species are mixed across the genus. To investigate this further, particularly to test the potential validity of the (synonymised) tri-forked genus ‘*Trictena*’, will require suitable genomic data (e.g., single nucleotide polymorphisms). This study now recognises the Geraldton Sandplains Region, covering 31,421 km^2^ of WA, as a region of high hepialid diversity, with 14 hepialid species, six of which are likely to be found predominantly nowhere else.

## Figures and Tables

**Table 1:** Checklist of all 52 species of *Abantiades* (including the five new species). The antennal forking structure (tines) indicated with no colour highlight for un-forked (Un), in green for bi-forked (bi), and in orange for tri-forked (tri). These grouped blocks to correspond to those used in Figure 2.

Species	Authority	Forking	Species	Authority	Forking
*A. albofasciatus*	(Swinhoe, 1892)	Un	*A. concordia*	Moore & Beaver, 2022	Bi
*A. antenniochrus*	Moore, 2014	Un	*A. karnka*	(Tindale, 1941)	Bi ^1^
*A. aphenges*	(Turner, 1904)	Un	*A. kolpodes* **sp. nov.**	Moore & Stevens	Bi
*A. argentangulum*	Moore & Edwards, 2014	Un	*A. lepusaures* **sp. nov.**	Moore & Stevens	Bi
*A. aurilegulus*	Tindale, 1932	Un	*A. mysteriella*	Simonsen, 2018	Bi
*A. barcas*	(Pfitzner, 1914)	Un	*A. paradoxa*	(Tindale, 1932)	Bi ^1^
*A. equipalpus*	Moore, 2014	Un	*A. pica* (‘Bordaia’-type)	(Tindale, 1932)	Bi ^1^
*A. fulvomarginatus*	Tindale, 1932	Un			
*A. furva*	(Tindale, 1932)	Un ^1^			
*A. hyalinatus* (type)	(Herrich-Schäffer, 1853)	Un	*A. argentata* (‘Trictina’-type)	(Tindale, 1932)	Tri ^2^
*A. hydrographus*	(R. Felder, 1874)	Un	*A. argyrosticha*	(Turner, 1929)	Tri ^2^
*A. labyrinthicus*	(Donovan, 1805)	Un	*A. atripalpis*	(Walker, 1856)	Tri ^2^
*A. latipennis*	Tindale, 1932	Un	*A. barnardi*	(Tindale, 1941)	Tri ^2^
*A. leucochiton*	(Pfitzner, 1914)	Un	*A. centralia*	Moore & Beaver, 2020	Tri
*A. lineacurva*	Moore & Edwards, 2014	Un	*A. cephalocorvus*	Moore & Beaver, 2020	Tri ^3^
*A. mcquillani*	Simonsen, 2018	Un	*A. horakae*	Simonsen, 2018	Tri
*A. magnificus*	(T.P. Lucas, 1898)	Un	*A. hutchinsoni*	Moore & Beaver, 2020	Tri
*A. malleus*	Moore & Beaver, 2022	Un	*A. incognito* **sp. nov.**	Moore & Stevens	Tri
*A. marcidus*	Tindale, 1932	Un	*A. inexpecta*	Simonsen, 2018	Tri ^3^
*A. moesta*	(Tindale, 1932)	Un^1^	*A. kayi*	Moore & Beaver, 2020	Tri
*A. neglecta*	Simonsen, 2018	Un	*A. kristenseni*	Simonsen, 2018	Tri ^3^
*A. ocellatus*	Tindale, 1932	Un	*A. macropusinsulariae*	Simonsen, 2018	Tri ^3^
*A. patella* **sp. nov.**	Moore & Stevens	Un	*A. obscura*	Simonsen, 2018	Tri
*A. penneshawensis*	Moore & Beaver, 2021	Un	*A. pallida*	Simonsen, 2018	Tri ^3^
*A. profundus* **sp. nov.**	Moore & Stevens	Un	*A. sui*	Simonsen, 2018	Tri
*A. rubrus*	Moore & Beaver, 2021	Un	*A. tembyi*	Moore & Beaver, 2020	Tri ^3^
*A. sericatus*	Tindale, 1932	Un	*A. zonatriticum*	Moore & Beaver, 2020	Tri

^1^ Un-forked and bi-forked species previously in the genus *Bordaia*. ^2^ Tri-forked species previously in the genus *Trictina*. ^3^ Tri-forked species united by ‘short-beaked’ genitalia morphology and mtDNA *COI* (compare to Figure 2).

## Data Availability

The data used in this study are openly available from GenBank (https://www.ncbi.nlm.nih.gov/genbank/, accessed on 20 February 2025) and our datasets are available from figshare (https://figshare.com/, accessed on 20 February 2025) at https://doi.org/10.6084/m9.figshare.31272334, accessed on 20 February 2025.

## Supplementary Materials

The following supporting information can be downloaded at: https://www.mdpi.com/article/10.3390/insects17030299/s1: Table S1. Specimen identification, Genbank accession numbers, collection/specimen data, for all specimens used in the morphological and molecular analyses (see Figure 2 and Table 1). Asterisk shows those previously in the genus *Bordaia*** and *Trictina****. Species united by ‘short-beaked’ genitalia morphology shown by a †. Table S2. Uncorrected patristic distances between all 58 sequences from 49 *Abantiades* species and the five outgroup taxa for the 658 bp mtDNA (*COI*) fragment. A single sequence is included for each species, except for the new species where we include all available sequences. The included Haplotypes are indicated by their codes (1–58). We did not have access to specimens or sequence data for *A. moesta* and *A. mysteriella*, and we were not able to generate useful sequence data for *A. incognito* sp. nov.

## Abbreviations

The following abbreviations are used in this manuscript: Australian states and territories: WA, Western Australia; SA, South Australia; Qld, Queensland; ACT, Australian Capital Territory; NT, Northern Territory. Collections: ANIC, Australian National Insect Collection, Canberra, ACT; SAMA, South Australian Museum, Adelaide, SA; WAM, Western Australian Museum, Perth, WA; QM, Queensland Museum, Brisbane Qld; RMNH, Naturalis Biodiversity Center Leiden, The Netherlands. Specimen label format (Material examined): Details on labels are verbatim for each specimen, except for detail in square brackets. Vertical lines | indicates line breaks on a label; after the first label, additional vertical lines indicates each additional label, such that || indicates a second label, and ||| indicates a third label, and so on.

## Appendix A

Below is the main terminology used in the main text for the three primary diagnostic characters used to identify *Abantiades* species: genitalia structure (Figure A1), antennal structure (Figure A2), and wing morphology (Figure A3).
